# GHSR‐Foxo1 Signaling in Macrophages Promotes Liver Fibrosis via Inflammatory Response and Hepatic Stellate Cell Activation

**DOI:** 10.1002/advs.202504223

**Published:** 2025-06-06

**Authors:** Da Mi Kim, Quan Pan, Zeyu Liu, Weiqi Ai, Hye Won Han, Sakhila K. Banu, Robert Y.L. Tsai, Gus A. Wright, Shaodong Guo, Yuxiang Sun

**Affiliations:** ^1^ Department of Nutrition Texas A&M University College Station TX 77843 USA; ^2^ Department of Veterinary Pathobiology Texas A&M University College Station TX 77843 USA; ^3^ Department of Veterinary Integrative Biosciences Texas A&M University College Station TX 77843 USA; ^4^ Institute of Biosciences and Technology Department of Translational Medical Sciences College of Medicine Texas A&M University Houston TX 77030 USA

**Keywords:** forkhead box protein o1 (Foxo1), growth hormone secretagogue receptors (GHSR), liver fibrosis, macrophages, transforming growth factor (TGF)‐β1

## Abstract

Liver fibrosis is a severe liver disease directly linked to chronic inflammation, in which hepatic macrophages play a key role. Growth hormone secretagogue receptor (GHSR) is the receptor of nutrient‐sensing hormone ghrelin that has essential functions in metabolism, inflammation, and wound‐healing. However, the role of GHSR in liver fibrosis is unknown. This study uses a carbon tetrachloride (CCl_4_)‐induced liver fibrosis mouse model to investigate the role of macrophage GHSR in liver fibrosis. CCl_4_ induces macrophage accumulation and inflammatory responses, noticeably increases GHSR expression in the liver. It is found that macrophage *Ghsr* deletion (*Ghsr‐MϕKO*) attenuates CCl_4_‐induced liver fibrosis and inflammation, showing reduced hepatic monocyte‐derived macrophages (MDMs) and suppressed proinflammatory responses. In macrophages, transforming growth factor (TGF)‐β1 expression is positively correlated with GHSR expression. GHSR‐associated TGF‐β1 in macrophages activates hepatic stellate cells (HSCs) by promoting the crosstalk between macrophages and HSCs. Macrophage GHSR controls inflammation and TGF‐β1 expression via protein kinase A (PKA)‐mediated Forkhead box protein O 1 (Foxo1) phosphorylation at S273; Foxo1‐S273D mutation, mimicking constitutive phosphorylation of Foxo1 at S273, shows exacerbated CCl_4_‐induced liver inflammation and fibrosis. Thus, targeting the macrophage GHSR−Foxo1 signaling may provide a new strategy to treat liver fibrosis.

## Graphical abstract

Macrophage GHSR‐Foxo1 axis regulates CCl4‐induced liver fibrosis by promoting inflammation and TGF‐β1‐mediated HSC activation. GHSR activates PKA‐dependent phosphorylation of Foxo1 at serine 273, promoting macrophage pro‐inflammatory polarization to enhance the production of pro‐inflammatory cytokines that damage hepatocytes, thereby inducing liver injury. Moreover, Foxo1 induces profibrotic cytokine, TGF‐β1, in macrophages, activating HSCs and driving excess ECM accumulation, eventually contributing to liver fibrosis.

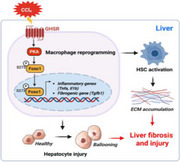

## 1 Introduction

Liver fibrosis, a pathological wound‐healing response with excessive accumulation of extracellular matrix (ECM), is a common pathology of many chronic liver diseases, including viral infection, alcohol abuse, hepatic toxicity, autoimmune conditions, and metabolic impairments.^[^ ^1^ ^]^ Liver fibrosis can lead to cirrhosis and hepatocellular carcinoma, causing significant mortality and economic burden.^[^ ^2^, ^3^, ^4^ ^]^ Upon profibrotic stimuli, proinflammatory mediators released by hepatic immune cells activate hepatic stellate cells (HSCs), which transdifferentiate into myofibroblasts; these myofibroblasts produce collagens and matrix degradation inhibitors, leading to excessive ECM deposition.^[^ ^5^ ^]^ Hepatic fibrosis is reversible in patients with chronic viral hepatitis, alcohol abuse, and metabolic dysfunction‐associated steatohepatitis, as well as in experimental rodent models of fibrosis, induced by Carbon tetrachloride (CCl_4_) administration or bile duct ligation.^[^ ^6^ ^]^ While the plastic nature of liver fibrosis may offer a therapeutic window of opportunity, effective antifibrotic therapies currently remain limited. Therefore, gaining a deeper understanding of the novel regulators and mechanisms underlying liver fibrosis is crucial for advancing the therapeutic options for liver fibrosis, as well as for preventing the progression of the disease.

Hepatic macrophages, the most abundant liver immune cells, are the major contributors to liver fibrosis.^[^ ^1^ ^]^ Liver macrophages comprise resident Kupffer cells (KCs) derived from erythromyeloid progenitors and infiltrating monocyte‐derived macrophages (MDMs) originated from the bone marrow.^[^ ^7^, ^8^ ^]^ Upon liver injury, KCs as early responders are activated, promoting liver inflammation by releasing proinflammatory cytokines such as tumor necrosis factor (TNF) α and interleukin (IL) 1β.^[^ ^9^ ^]^ At the same time, after liver injury, monocytes are recruited to the liver by chemokines such as CC‐chemokine ligand 2 (CCL2). Infiltrated monocytes in the liver are further differentiated into inflammatory, scar‐associated macrophages that serve as the major modulators of the fibrogenic process in chronic liver injury.^[^ ^10^ ^]^ These macrophages release fibrogenic factors, including transforming growth factor (TGF)‐β and platelet‐derived growth factor, which stimulate the transformation of quiescent HSCs into an activated form producing ECM, thus ultimately promoting liver fibrosis.

Growth hormone secretagogue receptor (GHSR), a G protein‐coupled receptor (GPCR), is recognized as the receptor for nutrient‐sensing hormone ghrelin. GHSR is mainly expressed in the central nervous system, particularly in the pituitary gland and the brain, but also in some peripheral organs such as the pancreas, spleen, kidneys, and adrenal glands.^[^ ^11^, ^12^, ^13^ ^]^ More importantly, we have shown that GHSR is expressed in macrophages, and its expression is increased in aging and high‐fat diet (HFD)‐induced obesity, and GHSR expression correlates with proinflammatory polarization of macrophages.^[^ ^14^, ^15^ ^]^ The Ghrelin−GHSR system is involved in growth hormone secretion, appetite regulation, fat accumulation, and energy expenditure.^[^ ^16^ ^]^ Due to the orexigenic properties of ghrelin, GHSR antagonists have been proposed for the treatment of obesity and diabetes. Ghrelin has been shown to have effects on injured livers, but the mediating cellular/molecular mechanism is an ongoing debate.^[^ ^17^, ^18^, ^19^, ^20^, ^21^ ^]^ We have demonstrated that ghrelin in monocytes is a crucial regulator of wound repair.^[^ ^22^ ^]^ Currently, it is unknown whether macrophage GHSR affects the pathogenesis and progression of liver fibrosis.^[^ ^23^ ^]^

Hepatic steatosis and inflammation are major risk factors for liver fibrogenesis. Our recent report demonstrated that in diet‐induced obese mice, myeloid GHSR deletion protects against hepatic steatosis and inflammation through protein kinase A (PKA)−cyclic adenosine monophosphate responsive element binding protein (CREB)−insulin receptor substrate 2 (IRS2) (PKA−CREB−IRS2) signaling cascade.^[^ ^15^ ^]^ In the present study, we used the chronic CCl_4_ treatment model to investigate the role of macrophage GHSR in liver fibrosis. We found that macrophage GHSR deletion reduces MDM infiltration, proinflammatory responses and liver fibrogenesis. Mechanistically, macrophage GHSR controls proinflammatory cytokine (TNFα and IL1β) and profibrotic cytokine (TGF‐β1) expression through transcription factor Forkhead box protein O1 (Foxo1), which in turn activates HSCs. Furthermore, we revealed that GHSR promotes Foxo1 and its target genes expression via PKA‐mediated phosphorylation at Ser 273 of Foxo1 (pFoxo1‐S273). These results indicate that the macrophage GHSR−Foxo1 nexus controls hepatic inflammation and fibrogenesis. GHSR, as a GPCR, is a favorable drug target, that may serve as a novel immunotherapeutic strategy for the treatment of liver fibrosis.

## 2 Results

### 2.1 Macrophage Inflammatory Responses are Upregulated in CCl_4_‐Induced Liver Fibrosis

**Figure 1:**
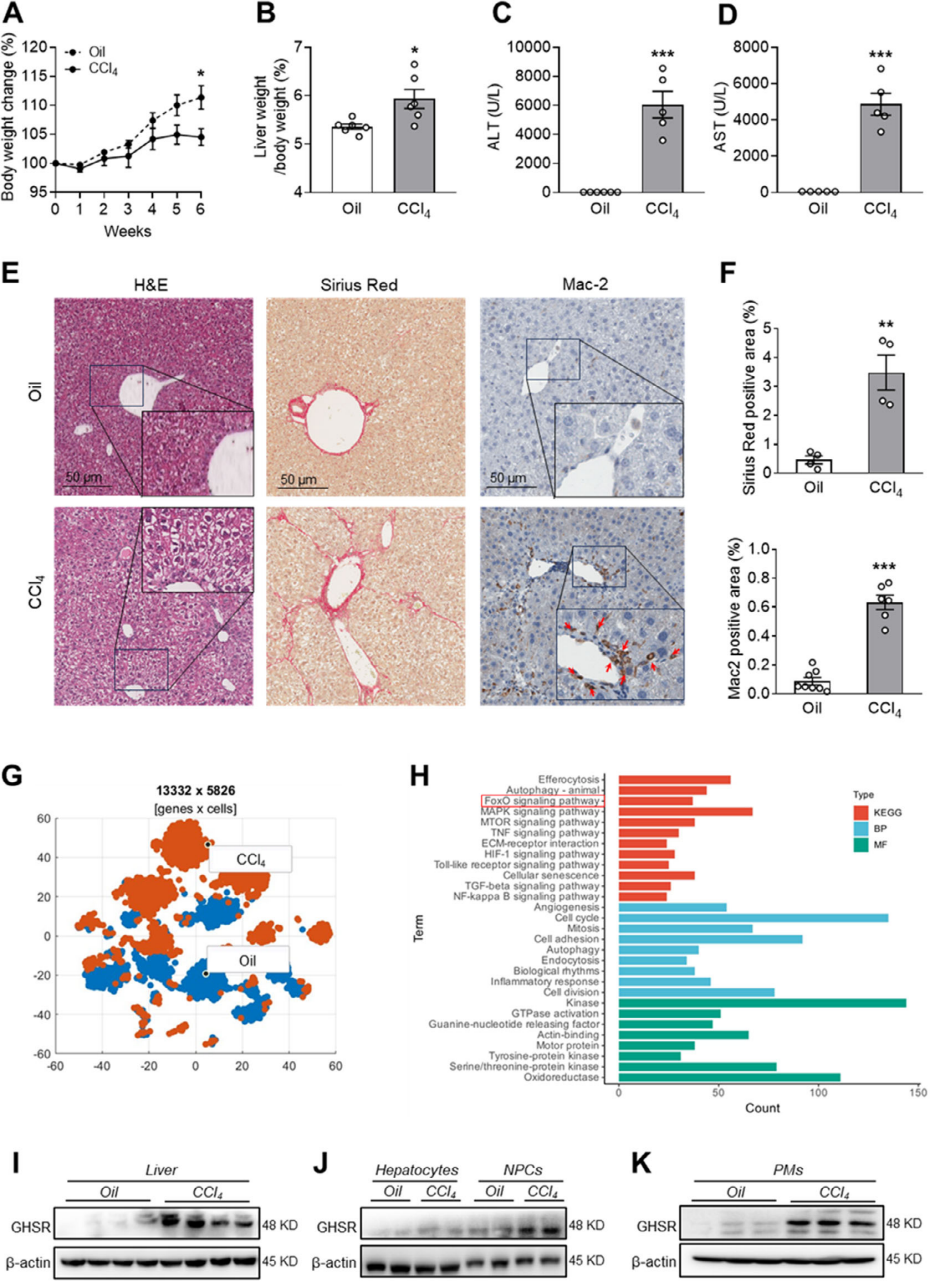
CCl_4_‐induced liver fibrosis is associated with increased macrophage infiltration and elevated inflammation in the liver. 8‐ to 12‐week‐old WT male mice were intraperitoneally (*i.p*.) injected with oil or CCl_4_ (2.5 µL g^−1^ body weight) twice per week for 6 weeks. A) Percentage of body weight change after CCl_4_ administration, *n* = 6 mice/group. B) Liver to body weight ratio, *n* = 6 mice/group. C and D) Serum ALT (C) and AST (D) levels, *n* = 5–6 mice/group. E) H&E, Sirius Red, and Mac‐2 staining of liver. Scale bar: 50 µm, *n* = 4 mice/group. F) Quantification of Sirius Red positive area and Mac‐2 positive area in Figure E. G and H) scRNAseq analysis of a public dataset GSE136103: (G) t‐SNE visualization of macrophages isolated from control (oil) and fibrotic (CCl_4_) mouse livers; (H) KEGG pathway, biological process (BP), molecular function (MF) analyses of the upregulated genes in macrophages in fibrotic livers versus control livers. I–K) GHSR protein expression in the liver (I), hepatocytes and non‐parenchymal cells (NPCs) (J), and peritoneal macrophages (PMs) (K), *n* = 2–4 mice/group. Data are presented as the means ± standard error of the means (SEM). *p < 0.05, **p < 0.01, ***p < 0.001 CCl_4_ versus oil using student *t*‐test.

To elucidate the effect of CCl_4_ on liver fibrosis and GHSR expression, we administered CCl_4_ to wild‐type (WT) mice and found that CCl_4_ reduced body weight but increased liver‐to‐body weight ratio (**Figure** **1**A,B). CCl_4_ administration profoundly elevated levels of serum Alanine aminotransferase (ALT) and Alanine aminotransferase (AST) (Figure 1C,D), caused hepatocyte ballooning, liver fibrosis, and increased macrophage infiltration; this was evident in the staining of Hematoxylin and eosin (H&E), Sirius Red, and Mac‐2, respectively (Figure 1E,F), suggesting the potential role of macrophages in the development of liver fibrosis. We next analyzed a published single‐cell RNA sequencing (scRNAseq) dataset (GSE136103) of macrophages from control (oil) and fibrotic (CCl_4_) mouse livers and found that the macrophage profile was significantly changed in the fibrotic livers (Figure 1G).^[^ ^10^ ^]^ Bioinformatics analysis, such as the Kyoto Encyclopedia of Genes and Genomes (KEGG) pathway and the Biological Process (BP), of upregulated differentially expressed genes (DEGs) in macrophages between oil and CCl_4_ groups demonstrated that CCl_4_ treatment activated inflammatory response‐related pathways, including Toll‐like receptor (TLR), TNF, and nuclear factor‐kappa B (NF‐*κ*B) (Figure 1H). Of note, FoxO signaling was significantly increased in the liver macrophages of CCl_4_ groups (Figure 1H). Our recent study revealed that GHSR, a GPCR that stimulates the activation of GTPase for signaling transduction, promotes macrophage inflammatory response and controls liver inflammation.^[^ ^15^ ^]^ Further molecular function (MF) analysis of these DEGs revealed the correlation of GTPase activation with liver fibrosis (Figure 1H), suggesting a potential activation of GHSR signaling by CCl_4_ treatment. We then measured the GHSR expression in the liver and found that GHSR protein levels were dramatically elevated by CCl_4_ (Figure 1I). Furthermore, we found that CCl_4_ treatment significantly increased GHSR protein levels in liver nonparenchymal cells (NPCs) but not in hepatocytes (Figure 1J). Due to the low fraction of liver macrophages in whole liver tissue, we detected the GHSR protein levels in peritoneal macrophages (PMs) of CCl_4_‐treated mice. We found that GHSR protein levels were markedly increased by CCl_4_ treatment in PMs (Figure 1K), suggesting that macrophage GHSR might be a key player in CCl_4_‐induced inflammatory responses. Taken together, these findings suggest that macrophage GHSR may be involved in controlling CCl_4_‐induced liver inflammation and fibrosis.

### 2.2 Macrophage *Ghsr* Deficiency Attenuates CCl_4_‐Induced Liver Inflammation, Injury, and Fibrosis

**Figure 2:**
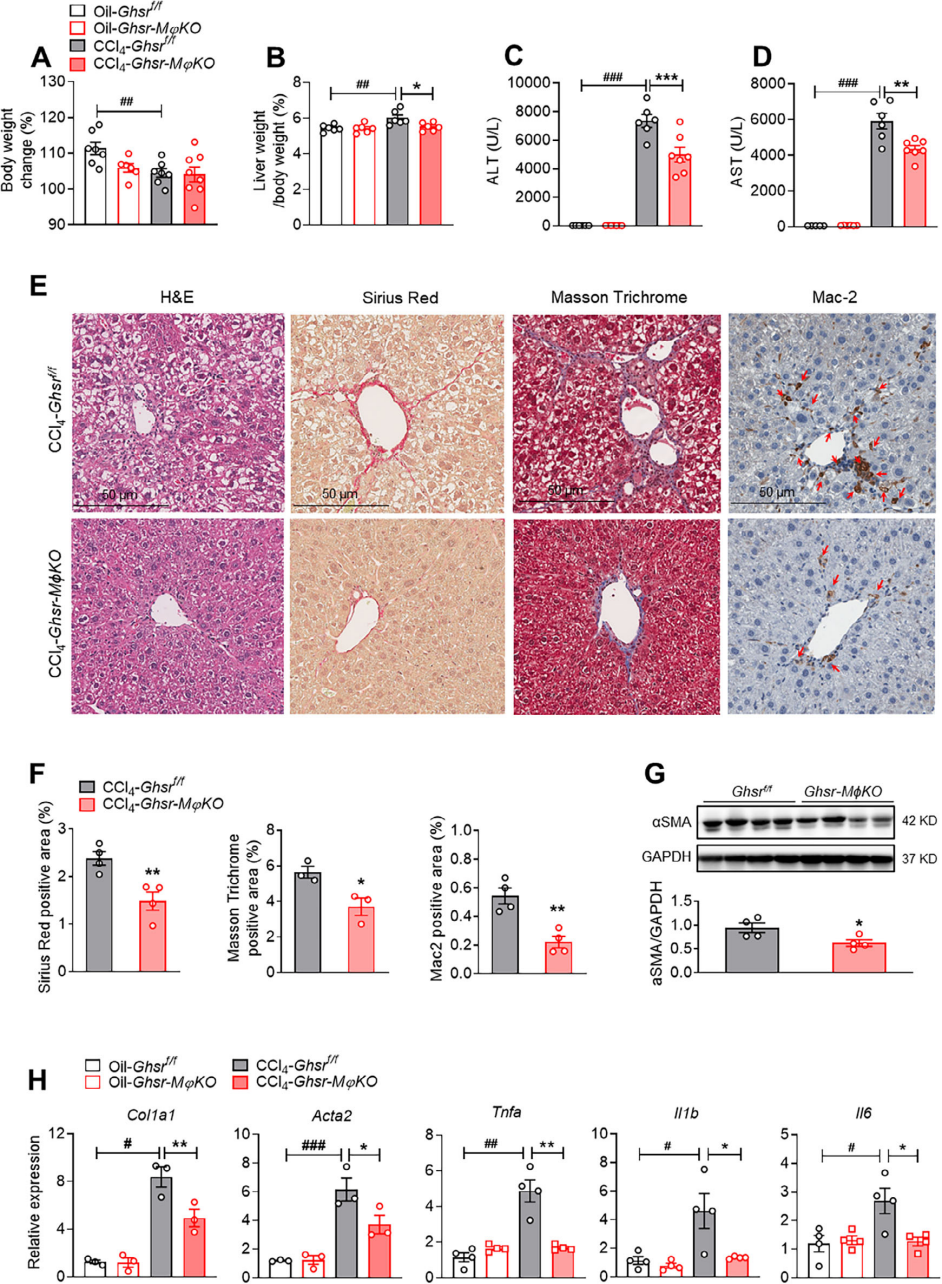
Macrophage *Ghsr* deficiency attenuates CCl_4_‐induced liver injury and fibrosis. 8‐ to 12‐week‐old *Ghsr‐MϕKO* male mice and their littermates (*Ghsr^f/f^*) were *i.p*. injected with oil or CCl_4_ (2.5 µL g^−1^ body weight) twice per week for 6 weeks. A and B) Percentage of body weight change (A) and liver/body weight ratio (B), *n* = 6–8 mice/group. C and D) Serum ALT (C) and AST (D) levels, *n* = 6–7 mice/group. E) H&E, Sirius Red, Masson's Trichrome, and Mac‐2 staining of livers. Scale bar: 50 µm, *n* = 3‐4 mice/group. F) Sirius Red positive area, Masson's Trichrome positive area, and Mac‐2 positive area, *n* = 3–4 mice/group. G) αSMA protein expression in the liver, *n* = 4 mice/group. H) Hepatic gene expression of fibrosis (*Col1a1*, *Acta2*) and proinflammatory cytokines (*Tnfa*, *Il1b*, *Il6*), *n* = 3–4 mice/group. Data are presented as the means ± SEM. *p < 0.05, **p < 0.01, ***p < 0.001 *Ghsr‐MϕKO* versus *Ghsr^f/f^*; ^#^ *P* < 0.05, ^##^ *P* < 0.01, ^###^ *P* < 0.001 CCl_4_ versus oil using Two‐way ANOVA.

To elucidate the role of macrophage GHSR in CCl_4_‐induced hepatic fibrosis, macrophage‐specific *Ghsr*‐deficient mice (*Ghsr‐MϕKO*) and control (*Ghsr^f/f^*) mice were injected with CCl_4_. Macrophage *Ghsr* deficiency abolished the CCl_4_‐induced alterations of body weight and liver/body weight ratio (**Figure** **2**A,B). Moreover, macrophage *Ghsr* deletion significantly reduced serum ALT and AST levels by 32.0% and 26.2%, respectively, in the CCl_4_‐treated mice (Figure 2C,D), suggesting a protective effect of macrophage *Ghsr* deficiency on CCl_4_‐induced liver injury. This was further supported by the evidence of reduced hepatocyte ballooning in the livers of *Ghsr‐MϕKO* mice (Figure 2E). Furthermore, macrophage *Ghsr* deficiency ameliorated CCl_4_‐induced liver fibrosis, indicated by significant decreases in Sirius Red staining, Masson Trichrome staining, and alpha‐smooth muscle actin (αSMA) protein levels in livers (Figure 2E–G), as well as reduced macrophage infiltration, indicated by the significant reduction of Mac‐2 staining (Figure 2E–G). Consistently, there was markedly reduced expression of fibrosis‐related genes, such as *Col1a1* and *Acta2* (encoding αSMA protein), and reduced expression of inflammatory cytokines including *Tnfa*, *Il1b*, and *Il6* in the livers of *Ghsr‐MϕKO* mice compared to those of control mice upon CCl_4_ treatment (Figure 2H). These findings support the protective effect of macrophage *Ghsr* deficiency on CCl_4_‐induced liver inflammation and suggest that the macrophage GHSR is an important contributor to hepatic inflammation and liver fibrosis.

### 2.3 Macrophage *Ghsr* Deficiency Attenuates the Infiltration and Proinflammatory Response of Hepatic Macrophages in CCl_4_‐Induced Liver Fibrosis

**Figure 3:**
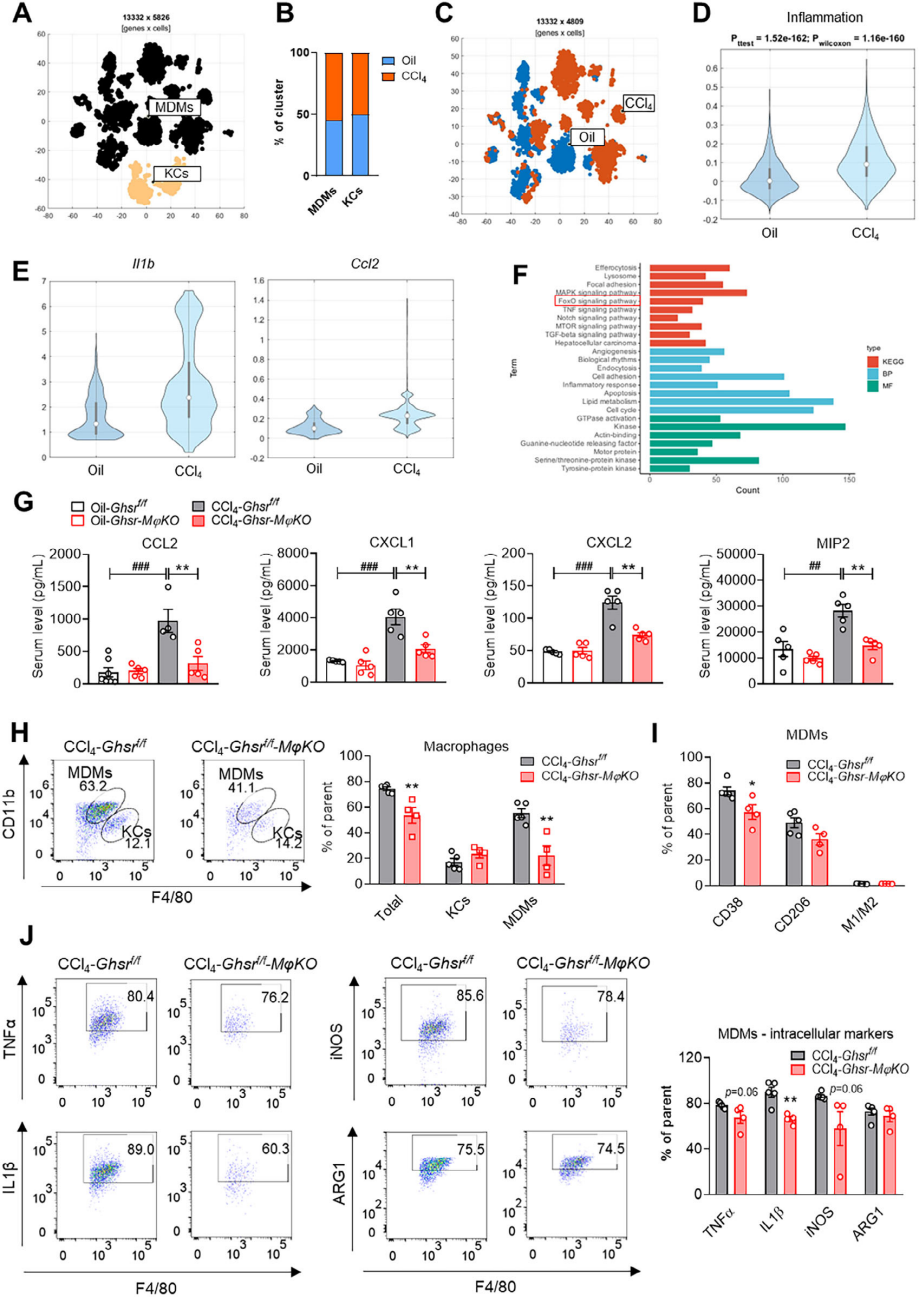
Macrophage *Ghsr* deficiency ameliorates hepatic inflammation and attenuates infiltration and proinflammatory activation of hepatic macrophages. A) t‐SNE visualization of monocyte‐derived macrophages (MDMs) and Kupffer cells (KCs) isolated from the livers of oil‐ or CCl_4_‐injected mice. B) Proportion of MDMs and KCs plotted as a stacked bar plot. C) t‐SNE visualization of MDMs isolated from the liver of oil and CCl_4_‐treated mice. D) Violin plot of inflammation score in MDMs from control and fibrotic livers. E) Violin plot of *Il1b* and *Ccl2* mRNA expression in MDMs from control and fibrotic livers. F) KEGG pathway, BP, MF analyses of the upregulated genes in MDMs in fibrotic livers versus control livers. G–J) 8‐ to 12‐week‐old *Ghsr‐MϕKO* male mice and their littermates were *i.p*. injected with oil or CCl_4_ (2.5 µL g^−1^ body weight) twice per week for 6 weeks. G) Serum chemokine levels, *n* = 4–7 mice/group. H) Percentage of MDM subset (CD45^+^Ly6G^−^CD11b^+^F4/80^low^) and KC subset (CD45^+^Ly6G^−^CD11b^+^F4/80^high^) of liver macrophages, *n* = 4–5 mice/group. I) M1‐ and M2‐related cell surface markers in MDMs. J) Intrinsic expression of inflammatory cytokines of TNFα, IL1β, iNOS, and ARG1, *n* = 4 mice/group. Data are presented as the means ± SEM. **p < 0.01 *Ghsr‐MϕKO* versus *Ghsr^f/f^*; ^##^ *P* < 0.01, ^###^ *P* < 0.001 CCl_4_ versus oil using Student *t*‐test or Two‐way ANOVA.

Hepatic macrophages, mainly comprised of tissue‐resident KCs and MDMs, play a key role in hepatic inflammation.^[^ ^24^ ^]^ scRNAseq analysis of macrophages from the livers of oil‐ and CCl_4_‐treated mice demonstrated an increase of MDMs population upon CCl_4_ treatment, but not of KCs (**Figure** **3**A–C; Figure S1A–C, Supporting Information).^[^ ^10^ ^]^ CCL2 is an essential chemokine that promotes macrophage infiltration, serving as a key chemoattractant to guide the macrophages to sites of inflammation or injury.^[^ ^25^ ^]^ This recruitment is largely driven by its interaction with the CC‐chemokine receptor 2 (CCR2) on macrophages. While *Ccl2* mRNA expression was increased in MDMs and KCs, both inflammation score and proinflammatory cytokine *Il1b* mRNA expression were significantly elevated by CCl_4_ in MDMs but not in KCs (Figure 3D,E; Figure S1C,D, Supporting Information).^[^ ^26^ ^]^ We next performed the KEGG pathway and BP analysis for the upregulated DEGs in the MDMs of CCl_4_‐injected mice and found that inflammatory response‐related pathways, such as TNF signaling, were activated in the MDMs of CCl_4_ group (Figure 3F). Further MF analysis of these DEGs revealed the activation of GTPase in MDMs of fibrotic liver. Our previous study demonstrated that macrophage *Ghsr* deletion reduced macrophage infiltration into liver and adipose tissue in diet‐induced obese mice.^[^ ^15^ ^]^ Given that macrophage *Ghsr* deletion significantly ameliorates hepatic inflammation, we then studied the effect of macrophage *Ghsr* deletion on liver macrophage infiltration and inflammatory responses. Interestingly, macrophage *Ghsr* deficiency profoundly ameliorated CCl_4_‐stimulated serum levels of chemokines, such as CCL2, C‐X‐C motif chemokine ligand (CXCL)1, CXCL2, and macrophage inflammatory protein (MIP)2 (Figure 3G), in line with reduced macrophage infiltration into liver during fibrosis. Indeed, flow cytometry analysis of hepatic macrophages further revealed *Ghsr* deletion resulted in a reduction of total macrophage population and MDMs population, but not of KCs (Figure 3H); this indicates that macrophage GHSR is involved in macrophage infiltration in response to liver injury. We further determined the effect of macrophage *Ghsr* deletion on polarization of MDMs. Macrophage *Ghsr* deletion significantly reduced CD38^+^ macrophages but not CD206^+^ macrophages (Figure 3I). Consistently, the expression of proinflammatory (M1) markers, including TNFα, IL1β, and iNOS, in the MDMs of macrophage *Ghsr*‐deficient mouse liver was reduced (Figure 3J), while antiinflammatory (M2) marker ARG1 in the MDMs was not affected (Figure 3J). These results indicate that GHSR is required for M1 polarization of MDMs, suggesting that macrophage GHSR contributes to hepatic inflammation via regulating macrophage infiltration and proinflammatory M1 polarization in liver fibrosis.

### 2.4 GHSR Controls TGF‐β1‐Mediated Crosstalk Between Macrophages and HSCs

**Figure 4:**
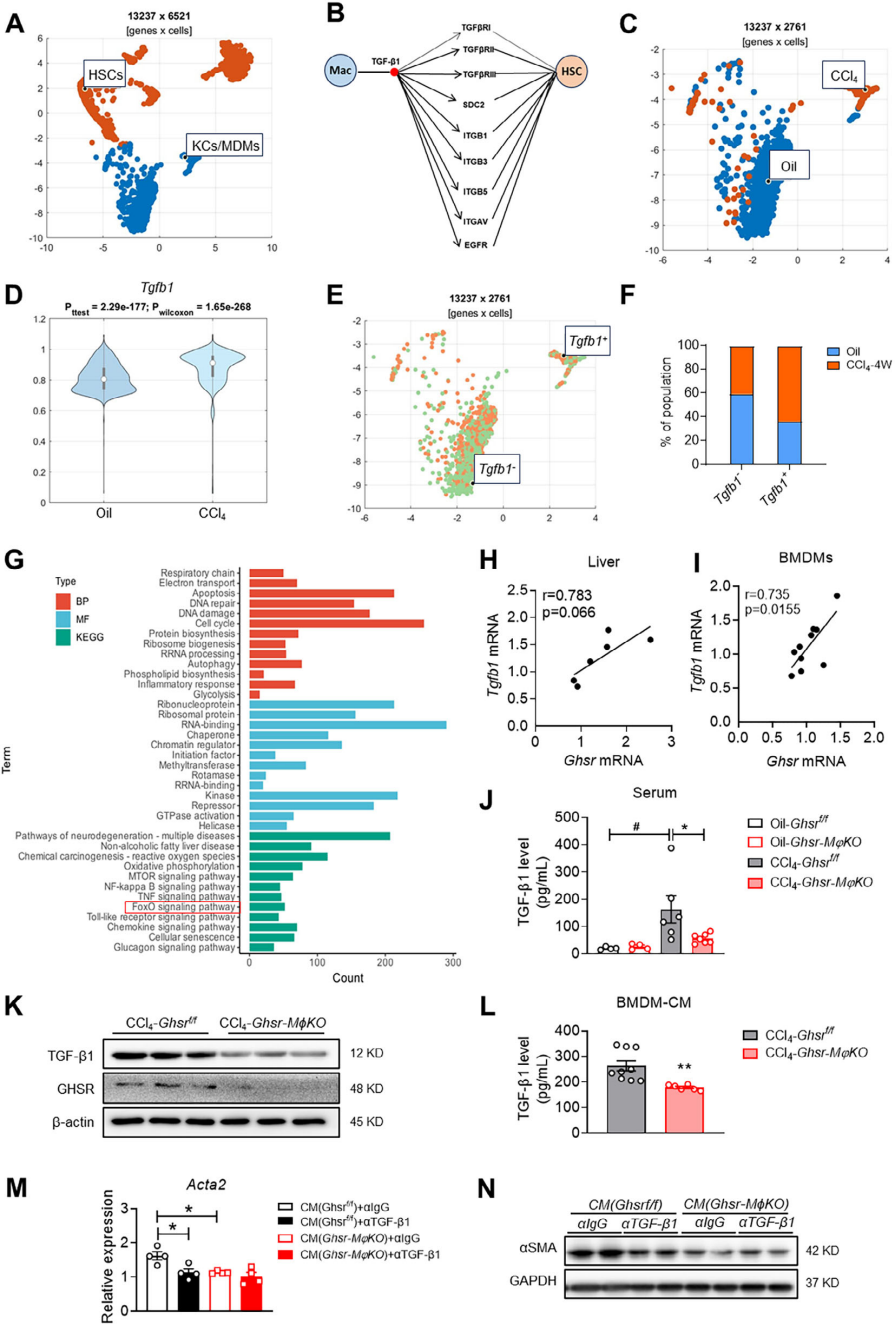
GHSR regulates TGF‐β1‐mediated crosstalk between macrophages and HSCs in CCl_4_‐induced liver fibrosis. A) U‐MAP visualization of macrophages and hepatic stellate cells (HSCs) isolated from the liver of mice injected with oil or CCl_4_. B) Predicted TGF‐β1 mediated crosstalk between macrophages and HSCs. C) U‐MAP visualization of macrophages shown in Figure 4A. D) Violin plot of *Tgfb1* expression in hepatic macrophages of mice injected with oil or CCl_4_. E) U‐MAP visualization of *Tgfb1* ^+^ and *Tgfb1* ^−^ macrophage clusters. F) Distribution of *Tgfb1* ^+^ and *Tgfb1* ^−^ macrophage clusters. G) KEGG pathway, BP, and MF analysis of marker genes in *Tgfb1* ^+^ macrophages. H and I) Correlations between the mRNA levels of *Ghsr* and *Tgfb1* in the livers (H) and bone marrow‐derived macrophages (BMDMs) (I). J) TGF‐β1 levels in serum of CCl_4_‐treated *Ghsr‐MϕKO* and *Ghsr^f/f^* mice, *n* = 4–7 mice/group. K) TGF‐β1 and GHSR protein expression in BMDMs of CCl_4_‐treated *Ghsr‐MϕKO* and *Ghsr^f/f^* mice, *n* = 3 mice/group. L) TGF‐β1 levels in conditioned medium (CM) collected from BMDMs (BMDM‐CM) isolated from CCl_4_‐treated *Ghsr‐MϕKO* and *Ghsr^f/f^* mice. (M and N) Primary HSCs were isolated from WT male mice. Cells were treated with BMDM‐CM isolated from CCl_4_‐treated *Ghsr‐MϕKO* and *Ghsr^f/f^* mice. CM was neutralized by TGF‐β1 antibody (α‐TGF‐β1) or control IgG. M) *Acta2* mRNA expression was determined by qPCR, *n* = 4/group. (N) αSMA protein expression was determined by Western blot, *n* = 2/group. Data are presented as the means ± SEM. *p < 0.05, **p < 0.01 *Ghsr‐MϕKO* versus *Ghsr^f/f^*; ^#^ *P* < 0.05 CCl_4_ versus oil using Student *t*‐test or Two‐way ANOVA.

The expression of TGF‐β1 in macrophages has a critical role in the pathogenesis of liver fibrosis.^[^ ^24^ ^]^ Indeed, scRNAseq analysis of liver NPCs (GSE145086) showed that there is a TGF‐β1‐mediated connection between macrophages and HSCs in CCl_4_‐induced liver fibrosis (**Figure** **4**A,B; Table S1, Supporting Information).^[^ ^27^ ^]^ In CCl_4_‐induced liver fibrosis, TGF‐β1 expression in liver macrophages was significantly elevated compared to that of controls (Figure 4C,D). We next clustered liver macrophages into two groups based on TGF‐β1 expression: *Tgfb1* ^+^ macrophages and *Tgfb1* ^−^ macrophages (Figure 4E), and found that CCl_4_ significantly increased *Tgfb1* ^+^ macrophages in the liver (Figure 4F). Further analysis of the marker genes of *Tgfb1* ^+^ macrophages indicated that these genes are associated with inflammatory response and GTPase activation (Figure 4G). Remarkably, we found there is a positive correlation between *Ghsr* and *Tgfb1* expression in the liver, and the association is more pronounced in bone marrow‐derived macrophages (BMDMs) (Figure 4H,I). The CCl_4_‐induced increases of circulating TGF‐β1 levels were significantly attenuated by macrophage *Ghsr* deletion (Figure 4J). Moreover, the expression and secretion of TGF‐β1 were significantly decreased in BMDMs from *Ghsr‐MϕKO* mice compared to those of control mice (Figures 4K,L). These results suggest that GHSR regulates TGF‐β1 expression in macrophages during liver fibrosis. To further elucidate the role of GHSR in the crosstalk between macrophages and HSCs, we isolated primary HSCs from control (*Ghsr^f/f^*) mice and treated them with conditioned medium (CM) from cultured BMDMs isolated from CCl_4_‐injected *Ghsr‐MϕKO* mice or control mice. HSCs treated with CM of *Ghsr‐MϕKO* BMDMs showed reduced fibrotic activation compared to those treated with CM of control BMDMs; this suggests that *Ghsr* deficiency mitigates the profibrotic effect of macrophages on HSCs (Figure 4M,N). Furthermore, TGF‐β1‐neutralized CM attenuated HSCs activation derived from control macrophages, but had no effect on HSCs activation derived from *Ghsr*‐deficient macrophages (Figure 4M,N). These results indicate that GHSR modulates TGF‐β1 expression of macrophages to promote the profibrotic effect of macrophages on HSC in the development of liver fibrosis.

### 2.5 GHSR Regulates the Expression of Proinflammatory and Profibrotic Cytokines in Macrophages via PKA‐Mediated Phosphorylation of Foxo1‐S273

**Figure 5:**
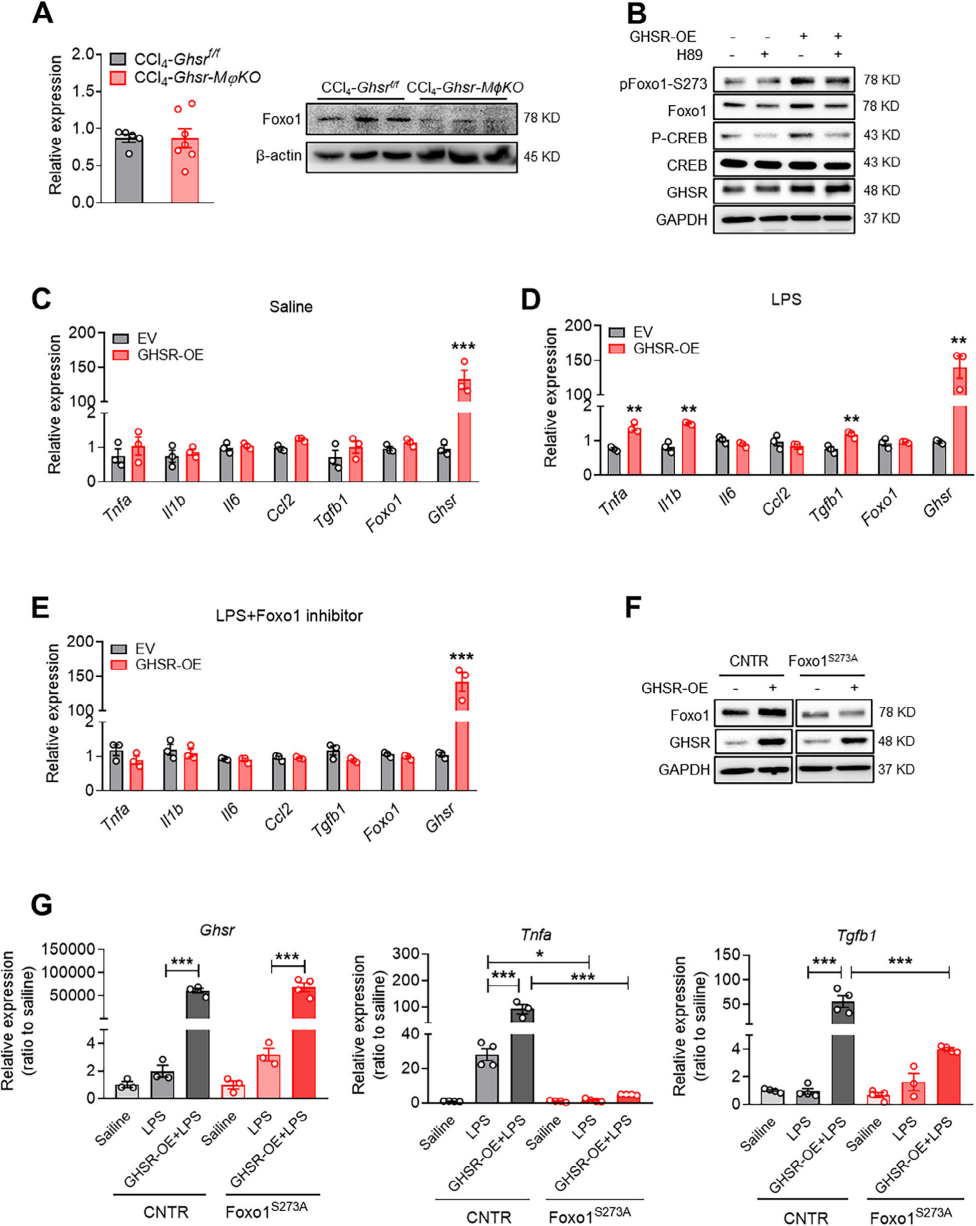
GHSR controls inflammatory responses and TGF‐β1 expression in macrophages via PKA‐mediated Foxo1‐S273 phosphorylation. A) Foxo1 mRNA and protein expression in BMDMs of mice injected with CCl_4,_ *n* = 3–7 mice/group (B) BMDMs were isolated from WT mice. Cells were transfected with GHSR overexpression (GHSR‐OE) plasmid or empty vector (EV) for 48 h and then treated with PKA inhibitor H89 (10 µM) for 4 h. Protein levels of pFoxo1‐S273, Foxo1, pCREB, CREB, and GHSR. C–E) BMDMs were isolated from WT mice. Cells were transfected with plasmid GHSR‐OE for 48 h, then examined mRNA expression of *Tnfa*, *Il1b*, *Il6*, *Ccl2*, *Tgfb1*, *Foxo1*, and *Ghsr* treated with saline (C), treated with LPS (100ng mL^−1^) for 4 h (D), or pretreated with Foxo1 inhibitor AS1842856 (10 µM) for 30 min, then treated with LPS (100 ng mL^−1^) for 4 h (E), *n* = 3/group. F) BMDMs were isolated from their littermate controls (CNTR) and Foxo1^S273A^ mice. Cells were transfected with plasmid GHSR‐OE for 48 h, and Foxo1 and GHSR protein levels were assessed by Western blotting. G) BMDMs were isolated from Foxo1^S273A^ mice and CNTR mice. Cells were transfected with plasmid GHSR‐OE for 48 h and then treated with LPS (100 ng mL^−1^) for 4 h. mRNA expression of *Ghsr, Tnfa*, and *Tgfb1*, *n* = 3–4/group. Data are presented as the means ± SEM. *p < 0.05, **p < 0.01, ***p < 0.001 GHSR OE versus EV using Student *t*‐test or One‐way ANOVA.

Macrophage Foxo1 has recently been shown to promote liver inflammation in mice with nonalcoholic steatohepatitis.^[^ ^28^ ^]^ In the current study, scRNAseq analysis indicated that Foxo1 signaling is activated in the macrophages of fibrotic livers and in *Tgfb1* ^+^ macrophages (Figures 1H and 4G). While *Foxo1* mRNA expression in macrophages was not altered by GHSR deletion, we observed a reduction of Foxo1 protein by GHSR deletion (**Figure** **5**A), suggesting a post‐transcriptional regulation of Foxo1 by GHSR. Our previous study revealed a post‐transcriptional regulation of Foxo1 by PKA signaling via phosphorylation at Serine 273 (pFoxo1‐S273).^[^ ^29^ ^]^ We have reported that GHSR promotes the inflammatory response in macrophages via activating PKA signaling.^[^ ^15^ ^]^ Therefore, we next examined whether macrophage GHSR regulates Foxo1 protein expression via PKA. Our results showed that the effect of GHSR‐induced Foxo1 protein expression was blocked by PKA inhibitor H89, suggesting that the GHSR−PKA pathway controls Foxo1 protein expression in macrophages (Figure 5B). To further determine whether GHSR exerts proinflammatory and profibrotic effects via Foxo1 signaling, we overexpressed GHSR in BMDMs and treated the cells with lipopolysaccharide (LPS), with or without Foxo1 inhibitor (Figure 5C–E). Compared to the Empty vector (EV) control group, GHSR overexpression (GHSR‐OE) robustly increased the expression of inflammatory cytokines, such as *Tnfa*, *Il1b*, and fibrogenic cytokine *Tgfb1*, under LPS stimulation (Figure 5D). Since the effects were blocked by Foxo1 inhibitor, the results support that GHSR promotes inflammatory response and fibrogenic effect via Foxo1 (Figure 5E). Next, we assessed the involvement of pFoxo1‐S273 in GHSR−PKA signaling in controlling the expression of Foxo1 protein and its activation. We found that pFoxo1‐S273 was dramatically increased by GHSR overexpression, while the effect was blocked by PKA inhibitor (Figure 5B). Moreover, GHSR overexpression‐induced Foxo1 protein expression was blocked in BMDMs with the Foxo1‐S273A mutation, which mimics Foxo1 dephosphorylation at Ser 273 (Figure 5F). These results suggest that GHSR promotes Foxo1 expression via PKA‐mediated phosphorylation at Foxo1‐S273. Furthermore, we overexpressed GHSR in BMDMs from S273A mice and their littermate controls (CNTR). We found that GHSR‐OE+LPS induced *Tnfa* and *Tgfb1* expression in CNTR cells, but not in S273A cells (Figure 5G). These observations indicate that GHSR promotes inflammatory responses and fibrogenic cytokine expression via PKA‐mediated Foxo1 phosphorylation at Ser 273.

### 2.6 Foxo1‐S273D Mutation Exacerbates CCl_4_‐Induced Liver Inflammation and Fibrosis

**Figure 6:**
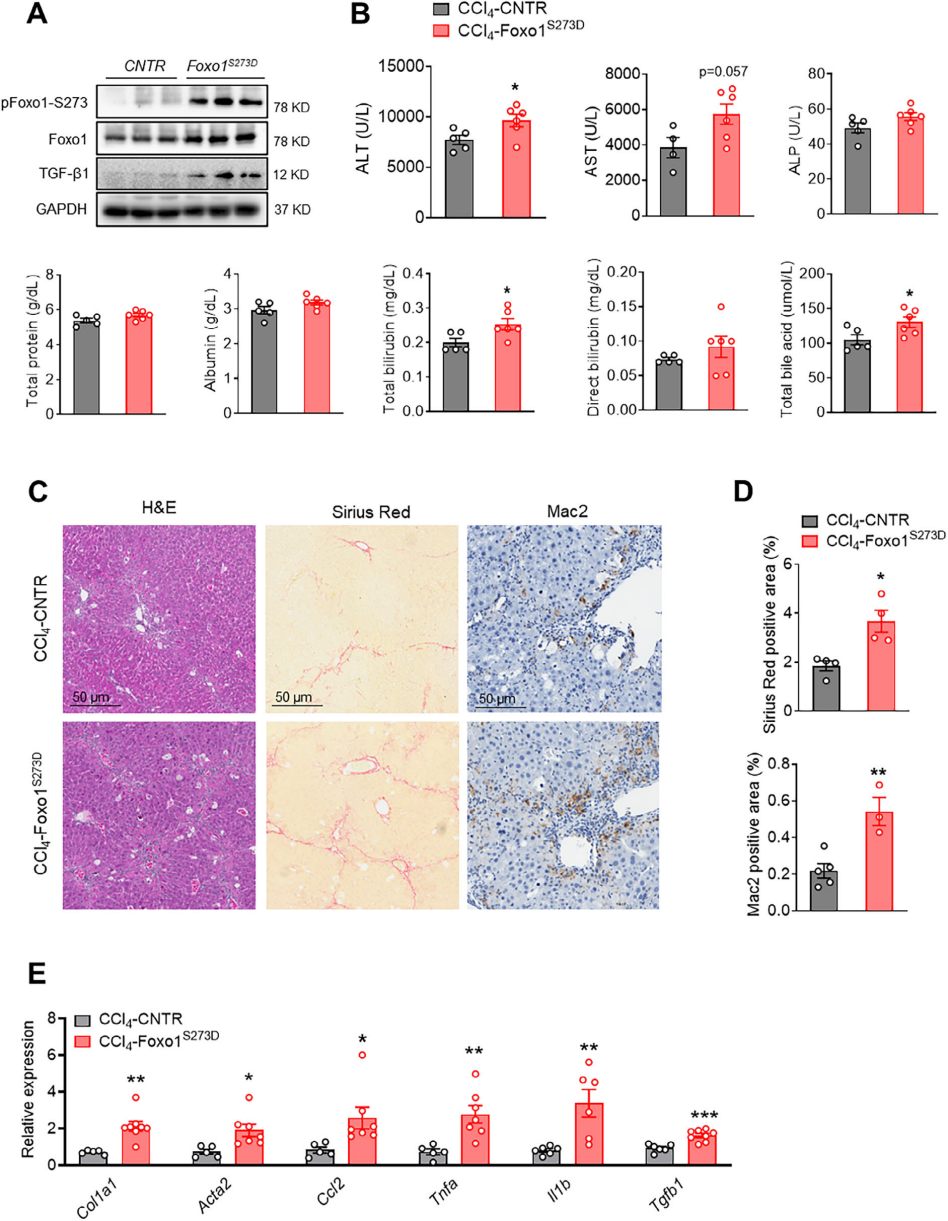
Foxo1^S273D^ mutation exacerbates CCl_4_‐induced liver fibrosis and inflammation. A) Protein levels of pFoxo1‐S273, and Foxo1 in BMDMs isolated of 8‐ to 12‐week‐old Foxo1^S273D^ male mice and CNTR mice, *n* = 3 mice/group. B–E) 8‐ to 12‐week‐old Foxo1^S273D^ male mice and CNTR mice were *i.p*. injected with CCl_4_ (2.5 µL g^−1^ body weight) twice per week for 6 weeks. B) Serum ALT, AST, alkaline phosphatase (ALP), total protein, albumin, total bilirubin, direct bilirubin, and total bile acid, *n* = 5–6 mice/group. C) H&E, Sirius Red, and Mac‐2 staining in livers. Scale bar: 50 µm, *n* = 4 mice/group. D) Sirius Red positive area and Mac‐2 positive area, *n* = 3–5 mice/group. E) mRNA expression of *Ghsr, Col1a1, Acta2, Ccl2, Tnfa, Il1b*, and *Tgfb1* in the livers, *n* = 5–7 mice/group. Data are presented as the means ± SEM. *p < 0.05, **p < 0.01 Foxo1^S273D^ versus CNTR using Student *t*‐test.

To further validate the role of pFoxo1‐S273 in liver inflammation and fibrosis, we injected CCl_4_ into Foxo1‐S273D mutant mice (Foxo1^S273D^), which mimics constitutive phosphorylation of Foxo1 at Ser 273. To validate constitutive phosphorylation of Foxo1 and Foxo1‐regulated TGF‐β1 expression in the macrophages of Foxo1^S273D^ mice, we isolated BMDMs and examined the protein levels of phosphorylated Foxo1‐S273 and TGF‐β1. Foxo1‐S273 phosphorylation was significantly enhanced in the BMDMs of Foxo1^S273D^ knockin mice compared to that of control mice (**Figure** **6**A). Accordingly, elevated TGF‐β1 protein expression along with increased Foxo1 protein levels in the BMDMs of Foxo1‐S273D mutation supports the regulation of TGF‐β1 by Foxo1 (Figure 6A). Constitutive activation of Foxo1 by S273D mutation exacerbated CCl_4_‐induced injury (Figure 6B). Foxo1^S273D^ mice exhibited more severe liver fibrosis and macrophage infiltration (Figure 6C,D). The mRNA expression of fibrotic genes of *Col1a1* and *Acta2*, inflammatory cytokines of *Tnfa* and *Il1b*, and chemokine *Ccl2* and cytokine *Tgfb1*, were increased in the livers of Foxo1^S273D^ mice (Figure 6E). Furthermore, increased hepatic *Tgfb1* gene expression observed in livers of CCl_4_‐injected Foxo1^S273D^ mice further confirms the role of Foxo1 in regulating TGF‐β1 in liver fibrosis. These findings indicate that Foxo1 phosphorylation at Ser 273 might serve as a checkpoint for the development of liver inflammation and fibrosis.

## 3 Discussion

Liver macrophages play a central role in driving hepatic inflammation in response to liver injury.^[^ ^24^ ^]^ Upon liver injury, damaged and dying cells rapidly release inflammatory mediators, including chemokines and cytokines to recruit leukocytes, to the injured sites. One of the earliest chemokines released after liver injury is CCL2 (also known as MCP‐1) which facilitates the recruitment of monocytes through its receptor, CCR2.^[^ ^24^ ^]^ Recruited monocytes differentiate into MDMs and polarize into a proinflammatory state to exacerbate hepatic inflammation. Our data suggest that the GHSR−Foxo1 network regulates both the recruitment and the inflammatory polarization of macrophages upon liver injury, based on the following findings: **1)** We observed a reduced hepatic macrophage population, especially infiltrated MDMs, in CCl_4_‐treated *Ghsr‐MϕKO* mice. CCl_4_‐induced increase of serum CCL2 levels was attenuated by macrophage *Ghsr* deletion. Foxo1^S273D^ mutant mice, in which Foxo1 is constitutively activated, exhibited exaggerated CCl_4_‐induced liver macrophage accumulation and *Ccl2* mRNA expression. Previous studies with myeloid Foxo1 knockout mice revealed the critical role of Foxo1 in control of *Ccl2* expression, and the recruitment of macrophages into tissues, such as liver and adipose tissue.^[^ ^28^, ^30^ ^]^ These results suggest that the GHSR−Foxo1 axis may regulate CCL2 to modulate the homing of monocytes into the liver; **2)** Foxo1 is an important mediator of insulin action that regulates cellular growth, metabolism, and survival via the PI3K−AKT cascade.^[^ ^31^ ^]^ Foxo1 is also recognized as a pivotal regulator of hepatic inflammation by controlling macrophage polarization.^[^ ^28^, ^32^, ^33^ ^]^ Consistently, our data demonstrated that macrophage Foxo1 signaling is activated during CCl_4_‐induced liver fibrosis. Our previous study showed that GHSR overexpression promotes IRS−AKT signaling, which is known to suppress Foxo1 activity by promoting its nuclear export and degradation.^[^ ^15^, ^34^ ^]^ Here, we found that GHSR overexpression stimulates Foxo1 protein expression in macrophages, which may be explained by the protective effect of PKA‐stimulated Foxo1‐S273 phosphorylation on AKT signaling‐induced Foxo1 degradation.^[^ ^29^ ^]^ Previously, Fan et al. demonstrated a TLR4−AKT−Foxo1 axis controlled self‐limiting mechanism by which macrophages avoid inappropriate overactivation after the initial inflammatory activation.^[^ ^33^ ^]^ During liver injury, the activation of GHSR−PKA−pFoxo1‐S273 signaling could impair the TLR4−AKT−Foxo1‐controlled self‐limiting inflammatory response, which may induce a state of persistent low‐grade inflammation that eventually leads to the development of liver fibrosis.

In addition to hepatotoxin, several other factors, such as obesity and aging, also cause chronic inflammation in the liver.^[^ ^1^ ^]^ In our previous study, GHSR expression is increased in macrophages under diet‐induced obesity; myeloid *Ghsr* deletion protects against hepatic inflammation and steatosis in obese mice.^[^ ^15^ ^]^ Interestingly, obese mice display an increased Foxo1 expression in hepatic macrophages, which inhibits macrophage anti‐inflammatory polarization via antagonizing STAT6 activity.^[^ ^28^ ^]^ During aging, macrophage GHSR and Foxo1 expression are also elevated.^[^ ^14^, ^32^ ^]^ *Ghsr* deletion reduced macrophage infiltration and proinflammatory cytokine expression in old mice.^[^ ^14^ ^]^ We showed that inhibiting Foxo1 activity reduces aging‐induced hepatic inflammation via attenuating inflammatory activation in KCs.^[^ ^32^ ^]^ These findings collectively suggest that targeting macrophage GHSR−Foxo1 signaling could be a novel strategy for combating chronic inflammation and associated injury.

TGF‐β1 is a key pathogenic driver of liver fibrosis by activating collagen‐producing HSCs in the injured liver.^[^ ^5^ ^]^ Although TGF‐β1 is ubiquitously expressed in all liver cells, scRNAseq analysis indicates that macrophages have the highest expression of TGF‐β1 in the liver (data not shown). TGF‐β1 secreted by macrophages is the strongest known activator of HSCs.^[^ ^35^ ^]^ Our current study showed that macrophage *Ghsr* deficiency significantly attenuated the increases of TGF‐β1 levels in serum, BMDMs, and BMDM‐CM induced by CCl_4_, strongly suggesting that GHSR plays a critical role in control of macrophage TGF‐β1 expression in liver injury and fibrosis. Mechanistically, our data suggest that GHSR promotes TGF‐β1 expression in macrophages by activating the PKA−Foxo1 signaling pathway. Indeed, Foxo1 is known to bind to the promoter region of *Tgfb1* gene and stimulates its expression.^[^ ^36^ ^]^ Previous study demonstrated that MDMs but not KCs are the major source of TGF‐β1 in the liver.^[^ ^37^ ^]^ Interestingly, our current data show that GHSR deletion reduces the population of MDMs but not KCs, suggesting that the reduced *Tgfb1* expression in the *Ghsr*‐deficient liver is likely due to reduced MDMs. The Foxo1‐regulated TGF‐β1 expression is also observed in hepatocytes. Hepatocyte Foxo1 deficiency reduces TGF‐β1 expression and attenuates CCl_4_‐induced liver fibrosis.^[^ ^38^ ^]^ Currently, it is unclear whether GHSR is also involved in the regulation of TGF‐β1 in hepatocytes. Nevertheless, targeting Foxo1 signaling could be a promising strategy for treating liver fibrosis. Indeed, inhibition of Foxo1 by either resveratrol or Foxo1 inhibitors have shown a protective effect in iron overload‐induced liver fibrosis.^[^ ^39^ ^]^ Moreover, anti‐oxidant Esculetin reduces TGF‐β1 expression and ameliorates HFD‐induced liver fibrosis in rats in a Foxo1‐dependent manner.^[^ ^40^ ^]^

Our study is the first report to elucidate the role of macrophage GHSR in liver fibrogenesis. GHSR is known as the receptor for ghrelin. Due to the orexigenic properties of ghrelin, GHSR antagonists have been proposed for the treatment of obesity and diabetes. Interestingly, in recent studies, ghrelin has been shown to exert antifibrotic effects in the injured liver, which raises the question of whether blockade of GHSR accelerates liver fibrosis, a liver disease commonly seen in patients with metabolic syndrome, obesity, and diabetes.^[^ ^17^, ^18^, ^19^, ^20^, ^21^ ^]^ Interestingly, our current study revealed a profibrotic effect of macrophage GHSR in chronic liver injury. Different mechanisms may explain the discrepant results of ligand ghrelin versus the macrophage‐specific effects of receptor GHSR. **First,** ligand‐dependent and ligand‐independent activities of GHSR have opposite effects on macrophage inflammatory response. In RAW264.7 macrophages, ghrelin inhibits the release of LPS‐induced proinflammatory cytokines by suppressing NF‐*κ*B signaling.^[^ ^41^ ^]^ Our recent study demonstrated that genetic GHSR deletion in macrophages suppresses LPS‐induced NF‐*κ*B signaling activation and the expression of proinflammatory cytokines in RAW264.7 macrophages and BMDMs.^[^ ^42^ ^]^ In addition, GHSR overexpression promotes LPS‐induced proinflammatory response in RAW264.7 macrophages and BMDMs.^[^ ^15^ ^]^ These results suggest that ligand‐dependent and ligand‐independent effects of GHSR may differ in macrophage inflammatory response. **Second,** the antifibrotic effect of ghrelin may be mediated by liver cells other than macrophages. Ghrelin has been reported to reduce TNFα‐induced hepatocyte apoptosis.^[^ ^43^ ^]^ Moreover, ghrelin reduces the expression of collagen I and TGF‐β1 in primary human HSCs.^[^ ^18^ ^]^ The beneficial effects of ghrelin in hepatocytes and HSCs could potentially attenuate fibrogenesis during liver injury. Future studies with hepatocyte‐ or HSC‐specific *Ghsr* knockout mice will help to clarify this question. **Third,** the antifibrotic effect of ghrelin may be independent of GHSR. In addition to macrophages, hepatocytes and activated HSCs have also been shown to express GHSR.^[^ ^18^ ^]^ However, it is unclear whether GHSR is involved in the beneficial effects of ghrelin in these cells. Indeed, several studies have shown that ghrelin also appears to be active in some tissues and cell lines that are not expressing GHSR.^[^ ^44^ ^]^ Nevertheless, our current study highlights the role of macrophage GHSR in control of liver fibrosis, which expands our knowledge of the pathogenesis of liver fibrosis.

We would like to acknowledge two limitations of the current study. The Foxo1‐S273D mutations were not myeloid‐specific but global. Given our previous finding that hepatocyte Foxo1 promotes CCl_4_‐induced hepatic inflammation and fibrosis.^[^ ^38^ ^]^ The exacerbated fibrosis development in Foxo1^S273D^ mice may also be due to the activation of Foxo1 in hepatocytes. Further scRNAseq analysis of liver cells from Foxo1^S273D^ mutant mice would be beneficial to define the effects of Foxo1‐S273 phosphorylation in different liver cells, such as hepatocytes and NPCs, in liver fibrosis. Nevertheless, our in vitro analysis using BMDMs from Foxo1^S273A^ mice supports a cell‐autonomous role of Foxo1‐S273 phosphorylation in mediating GHSR‐driven macrophage polarization and TGF‐β1 expression, which is at least partially involved in the fibrosis development. Also, our current study was conducted exclusively in male mice. Given that female hormone estrogen suppresses Foxo1 activity, future studies with female mice would be important to determine whether our findings apply to both sexes.^[^ ^45^ ^]^

## 4 Conclusion

In summary, our study demonstrates the novel role of nutrient‐sensing macrophage GHSR in liver fibrosis, the GHSR−Foxo1 nexus is an important regulatory pathway in liver macrophages to inflammation and fibrosis. Mechanistically, GHSR controls the expression of proinflammatory cytokines of *Tnfa*, *Il1b*, and fibrogenic cytokine *Tgfb1* in macrophages, which collectively exert paracrine effects on HSCs to promote HSC activation and fibrogenesis. The proinflammatory and profibrogenic effects of GHSR are driven by Foxo1, specifically through PKA‐mediated phosphorylation at Serine 273. Our findings suggest that suppressing GHSR signaling in macrophages may serve as a novel therapeutic strategy for liver fibrosis.

## 5 Experimental Section

### Animals

The floxed *Ghsr* (*Ghsr^f/f^*) mice were generated as described previously.^[^ ^15^ ^]^ To generate myeloid‐specific *Ghsr*‐deficient mice, *Ghsr^f/f^* mice were crossed with LysM‐Cre mice (JAX stock 4781) and backcrossed on C57/BL6 background. Foxo1^S273A^ and Foxo1^S273D^ knock‐in (KI) mice were generated as described previously.^[^ ^29^ ^]^ Foxo1^S273A^ and Foxo1^S273D^ mice have been fully backcrossed on C57/BL6 background. All mice used in this study were males at 8∼12 weeks of age and were randomly assigned to either cohort or CCI_4_ treatment groups. All mice were housed at 24⁰C in a 12/12‐hr light‐dark cycle, and were given free access to food (regular diet, Teklad 2018) and water *at libitum*. All animal protocols were approved by the Institutional Animal Care and Use Committee of Texas A&M University (Approval number: IACUC 2022‐0100, IACUC 2022‐0180).

### CCl_4_ Injection

To induce liver fibrosis by CCl_4_, mice were given corn oil or 20% solution of CCl_4_ (2.5 µL g^−1^ body weight i.p. injection twice per week) for 6 weeks, and then euthanized for tissue harvest 72 h after the last CCl_4_ injection.

### Serum Biochemistry Analysis

AST, ALT, ALP, total protein, albumin, total bilirubin, direct bilirubin, and total bile acid were measured using DxC 700 AU Chemistry Analyzer (Beckman Coulter, Brea, CA) at the Texas A&M preclinical phenotyping core. Serum cytokine levels and TGF‐β1 levels were measured using a commercially available kit (Millipore‐Sigma, Burlington, MA) with a Multiplex Reader (Bio‐Rad, Hercules, CA) and Luminex Reader (BMG LABTECH, Ortenberg, Germany), according to the manufacturers’ instructions, respectively.

### Histology

Mouse liver samples were fixed in 10% neutral buffered formalin, then embedded into paraffin blocks, sectioned at 5 microns, and stained with H&E, Sirius Red, or Masson Trichrome, antibody against Mac‐2 (Abcam, Cambridge, United Kingdom) according to routine immunohistochemistry protocols.^[^ ^15^ ^]^ Percentage of Sirius Red positive areas and Mac‐2 positive areas in liver sections were analyzed using Image J software (Tree Star Inc., Ashland, OR).

### Isolation and Flow Cytometry Analysis of Peritoneal Macrophages and NPCs of Liver

Peritoneal macrophages and liver NPCs were obtained from the mice as previously described.^[^ ^32^, ^46^ ^]^ Cells were collected for flow cytometry analysis on Cytek® Aurora (Cytek, Fremont, CA) and analyzed using FlowJo software (Tree Star Inc.).

### Isolation, Culture, and Treatment of BMDMs

Bone marrow cells were isolated from the tibias and femurs of mice as described.^[^ ^15^, ^47^ ^]^ Cells were transfected with GHSR overexpression plasmid pcDNA3.1‐*Ghsr* which is constructed by cloning the coding region of *Ghsr* into a pcDNA3.1 vector (Invitrogen, Waltham, MA) as previously described.^[^ ^48^ ^]^ After 48 h of transfection, cells were treated with PKA inhibitor H89 (10 µM) or Foxo1 inhibitor AS1842856 (10 µM) for 30 min, followed by LPS treatment (10 ng mL^−1^) for 4 h, and then collected for quantitative real‐time PCR (qPCR) and Western blotting analysis.

### Isolation, Culture, and Treatment of Mouse Primary HSCs

Mouse primary HSCs were isolated as described previously.^[^ ^49^ ^]^ Cells were treated with CM collected from BMDMs with or without TGF‐β1 neutralization by TGF‐β1 antibody (0.5 µg mL^−1^) (ThermoFisher Scientific) for 12 h.

### Quantitative Real‐time PCR

Total RNA isolation, cDNA synthesis, quantitative real‐time PCR reaction, and data analysis were performed as previously described.^[^ ^38^ ^]^ See Table S2 for the primers’ information (Supporting Information).

### Western Blotting

Proteins were extracted from BMDMs and adjusted to equal amounts for Western blotting as described.^[^ ^15^ ^]^ Antibody for pFoxo1‐S273 was generated as previously described.^[^ ^29^ ^]^ Antibodies for Foxo1, TGF‐β1, p‐CREB, CREB, β‐actin, and GAPDH were purchased from Cell Signaling Technology, αSMA from Abcam, and GHSR from Invitrogen (see Table S2, Supporting Information). The signal intensity was visualized with ECL (Genesee Scientific, Morrisville, NC) and analyzed using NIH Image J software.

### Analysis of scRNAseq Datasets

Two public scRNAseq datasets were analyzed:GSE136103 (scRNAseq of macrophages isolated from control (oil) and fibrotic (CCl_4_) mouse livers) and GSE145086 (scRNAseq of NPCs isolated from oil or CCl_4_‐treated mouse livers).^[^ ^10^, ^27^ ^]^ The t‐SNE, plots, UMAP plots, violin plots, and bar plots were generated by scGEAToolbox in MATLAB as described.^[^ ^50^ ^]^

### Statistical Analysis

All data were presented as mean ± standard error of the mean. Statistical analyses were performed with GraphPad Prism 8.01. Student's *t*‐test was used to determine the significance of the differences between two groups. One‐way or Two‐way ANOVA test was used to determine the significance of the differences between multiple groups.; *P < 0.05 is considered statistically significant; **p < 0.01; ***p < 0.001 shows more pronounced differences. All authors had access to the study data and had reviewed and approved the final manuscript.

## Conflict of Interest

The authors declare no conflict of interest.

## Author Contributions

D.M.K. and Q.P. contributed equally to this work as co‐first authors. S.G. and Y.S. are the co‐corresponding authors. D.M.K. and Q.P.: conceptualization, formal analysis, methodology, data curation, investigation, visualization, and writing original draft; Z.L. and W.A.: investigation and data curation; H.W.H.: methodology; S.K.B.: writing review & editing; R.T. and G.A.W.: formal analysis and writing review & editing; S.G. and Y. S.: funding acquisition, supervision, and writing review & editing.

## Supporting information



Supporting Information

## Data Availability

The data that support the findings of this study are available from the corresponding author upon reasonable request.

## References

[1] H. Gilgenkrantz , R. A. Sayegh , S. Lotersztajn , Annu. Rev. Pharmacol. Toxicol. 2025, 65, 1. 39259981 10.1146/annurev-pharmtox-020524-012013

[2] Z. M. Younossi , G. Wong , Q. M. Anstee , L. Henry , Clin. Gastroenterol. Hepatol. 2023, 21, 1978. 37121527 10.1016/j.cgh.2023.04.015

[3] H. Devarbhavi , S. K. Asrani , J. P. Arab , Y. A. Nartey , E. Pose , P. S. Kamath , J. Hepatol. 2023, 79, 516. 36990226 10.1016/j.jhep.2023.03.017

[4] F. Piscaglia , G. Svegliati‐Baroni , A. Barchetti , A. Pecorelli , S. Marinelli , C. Tiribelli , S. Bellentani , Hepatology 2016, 63, 827.26599351

[5] R. Bataller , D. A. Brenner , J. Clin. Invest. 2005, 115, 209. 15690074 10.1172/JCI24282 PMC546435

[6] T. Kisseleva , D. Brenner , Nat. Rev. Gastroenterol. Hepatol. 2021, 18, 151. 33128017 10.1038/s41575-020-00372-7

[7] O. Krenkel , F. Tacke , Nat. Rev. Immunol. 2017, 17, 306. 28317925 10.1038/nri.2017.11

[8] E. Gomez Perdiguero , K. Klapproth , C. Schulz , K. Busch , E. Azzoni , L. Crozet , H. Garner , C. Trouillet , M F. de Bruijn , F. Geissmann , H.‐R. Rodewald , Nature 2015, 518, 547. 25470051 10.1038/nature13989 PMC5997177

[9] Y. Wen , J. Lambrecht , C. Ju , F. Tacke , Cell Mol. Immunol. 2021, 18, 45. 33041338 10.1038/s41423-020-00558-8 PMC7852525

[10] P. Ramachandran , R. Dobie , J. R. Wilson‐Kanamori , E. F. Dora , B. E. P. Henderson , N. T. Luu , J. R. Portman , K. P. Matchett , M. Brice , J. A. Marwick , R. S. Taylor , M. Efremova , R. Vento‐Tormo , N. O. Carragher , T. J. Kendall , J. A. Fallowfield , E. M. Harrison , D. J. Mole , S. J. Wigmore , P. N. Newsome , C. J. Weston , J. P. Iredale , F. Tacke , J. W. Pollard , C. P. Ponting , J. C. Marioni , S. A. Teichmann , N. C. Henderson , Nature 2019, 575, 512. 31597160 10.1038/s41586-019-1631-3 PMC6876711

[11] X.‐M. Guan , H. Yu , O. C. Palyha , K. K. McKee , S. D. Feighner , D. J. S. Sirinathsinghji , R G. Smith , L. H. T. Van der Ploeg , A. D. Howard , Mol. Brain Res. 1997, 48, 23. 9379845 10.1016/s0169-328x(97)00071-5

[12] S. Gnanapavan , B. Kola , S. A. Bustin , D. G. Morris , P. McGee , P. Fairclough , S. Bhattacharya , R. Carpenter , A. B. Grossman , M. Korbonits , The J. Clin. Endocrinol. Metabol. 2002, 87, 2988. 10.1210/jcem.87.6.8739 12050285

[13] X. Xiao , M. Bi , Q. Jiao , X. Chen , X. Du , H. Jiang , Ageing Res. Rev. 2020, 64, 101187. 33007437 10.1016/j.arr.2020.101187

[14] L. Lin , J. H. Lee , E. D. Buras , K. Yu , R. Wang , C. W. Smith , H. Wu , D. Sheikh‐Hamad , Y. Sun , Aging (Albany NY) 2016, 8, 178. 26837433 10.18632/aging.100888 PMC4761721

[15] Da Mi Kim , J. H. Lee , Q. Pan , H. W. Han , Z. Shen , S. Eshghjoo , C.‐S. Wu , W. Yang , Ji Y Noh , D W. Threadgill , S. Guo , G. Wright , R. Alaniz , Y. Sun , Mol. Metab. 2024, 79, 101852. 38092245 10.1016/j.molmet.2023.101852 PMC10772824

[16] M. A. Hedegaard , B. Holst , Endocrinology 2020, 161, bqaa020. 32049280 10.1210/endocr/bqaa020

[17] Y. Mao , S. Zhang , F. Yu , H. Li , C. Guo , X. Fan , Int. J. Mol. Sci. 2015, 16, 21911. 26378522 10.3390/ijms160921911 PMC4613288

[18] M. Moreno , J. F. Chaves , P. Sancho‐Bru , F. Ramalho , L. N. Ramalho , M. L. Mansego , C. Ivorra , M. Dominguez , L. Conde , C. Millán , M. Marí , Hepatology 2010, 51, 974. 20077562 10.1002/hep.23421

[19] Y. Li , J. Hai , L. Li , X. Chen , H. Peng , M. Cao , Q. Zhang , Endocrine 2013, 43, 376. 22843123 10.1007/s12020-012-9761-5

[20] S. Ezquerro , C. Tuero , S. Becerril , V. Valentí , R. Moncada , M. F. Landecho , V. Catalán , J. Gómez‐Ambrosi , F. Mocha , C. Silva , K. P. Hanley , Eur. J. Endocrinol. 2023, 189, 1. 10.1093/ejendo/lvad071 37358209

[21] J. Zhu , T. Zhou , M. Menggen , K. Aimulajiang , H. Wen , Front. Cell. Infection Microbiol. 2024, 13, 1324134. 10.3389/fcimb.2023.1324134 PMC10800934 38259969

[22] R. M. Kratofil , H. B. Shim , R. Shim , W. Y. Lee , E. Labit , S. Sinha , C. M. Keenan , B. G. Surewaard , J. Y. Noh , Y. Sun , K. A. Sharkey , Nature 2022, 609, 166. 35948634 10.1038/s41586-022-05044-x

[23] P. Angulo , J. C. Keach , K. P. Batts , K. D. Lindor , Hepatology 1999, 30, 1356. 10573511 10.1002/hep.510300604

[24] L. Hammerich , F. Tacke , Nat. Rev. Gastroenterol. Hepatol. 2023, 20, 633. 37400694 10.1038/s41575-023-00807-x

[25] C. Shi , E. G. Pamer , Nat. Rev. Immunol. 2011, 11, 762. 21984070 10.1038/nri3070 PMC3947780

[26] N. Yuan , Y. Chen , Y. Xia , J. Dai , C. Liu , Transl. Psychiat. 2019, 9, 233. 10.1038/s41398-019-0570-y PMC6751188 31534116

[27] M. K. Terkelsen , S. M. Bendixen , D. Hansen , E. A. Scott , A. F. Moeller , R. Nielsen , S. Mandrup , A. Schlosser , T. L. Andersen , G. L. Sorensen , A. Krag , Hepatology 2020, 72, 2119. 32145072 10.1002/hep.31215 PMC7820956

[28] S. Lee , T. O. Usman , J. Yamauchi , G. Chhetri , X. Wang , G. M. Coudriet , C. Zhu , J. Gao , R. McConnell , K. Krantz , D. Rajasundaram , J. Clin. Invest. 2022, 132, 154333. 10.1172/JCI154333 PMC9282937 35700043

[29] Y. Wu , Q. Pan , H. Yan , K. Zhang , X. Guo , Z. Xu , W. Yang , Y. Qi , C A. Guo , C. Hornsby , L. Zhang , A. Zhou , L. Li , Y. Chen , W. Zhang , Y. Sun , H. Zheng , F. Wondisford , L. He , S. Guo , Diabetes 2018, 67, 2167. 30201683 10.2337/db18-0674 PMC6198346

[30] D. Xu , X. Qu , T. Yang , M. Sheng , X. Bian , Y. Zhan , Y. Tian , Y. Lin , Y. Jin , X. Wang , M. Ke , Exp. Mol. Med. 2024, 56, 1843. 39122845 10.1038/s12276-024-01280-5 PMC11372114

[31] D. Accili , K. C. Arden , Cell 2004, 117, 421. 15137936 10.1016/s0092-8674(04)00452-0

[32] W. Yang , D. M. Kim , W. Jiang , W. Ai , Q. Pan , S. Rahman , J. J. Cai , W. A. Brashear , Y. Sun , S. Guo , Aging Cell 2023, 22, 13968. 10.1111/acel.13968 PMC10577549 37602516

[33] W. Fan , H. Morinaga , J. J. Kim , E. Bae , N. J. Spann , S. Heinz , C. K. Glass , J. M. Olefsky , EMBO J. 2010, 29, 4223. 21045807 10.1038/emboj.2010.268 PMC3018786

[34] S. Guo , G. Rena , S. Cichy , X. He , P. Cohen , T. Unterman , J. Biol. Chem. 1999, 274, 17184. 10358076 10.1074/jbc.274.24.17184

[35] Y. Koyama , D. A. Brenner , J. Clin. Invest. 2017, 127, 55. 28045404 10.1172/JCI88881 PMC5199698

[36] Q. Pan , W. Ai , Y. Chen , Da Mi Kim , Z. Shen , W. Yang , W. Jiang , Y. Sun , S. Safe , S. Guo , Diabetes 2023, 72, 1193. 37343276 10.2337/db23-0180 PMC10450826

[37] K. R. Karlmark , R. Weiskirchen , H W. Zimmermann , N. Gassler , F. Ginhoux , C. Weber , M. Merad , T. Luedde , C. Trautwein , F. Tacke , Hepatology 2009, 50, 261. 19554540 10.1002/hep.22950

[38] Q. Pan , M. Gao , D. Kim , W. Ai , W. Yang , W. Jiang , W. Brashear , Y. Dai , S. Li , Y. Sun , Y. Qi , S. Guo , Cell. Mol. Gastroenterol. Hepatol. 2024, 17, 41. 37678798 10.1016/j.jcmgh.2023.08.013 PMC10665954

[39] S K. Das , W. Wang , P. Zhabyeyev , R. Basu , B. McLean , D. Fan , N. Parajuli , J. DesAulniers , V B. Patel , R J. Hajjar , J R. B. Dyck , Z. Kassiri , G Y. Oudit , Sci. Rep. 2015, 5, 18132. 26638758 10.1038/srep18132 PMC4671148

[40] A. Pandey , P. Raj , S. K. Goru , A. Kadakol , V. Malek , N. Sharma , A. B. Gaikwad , Pharmacolog. Rep. 2017, 69, 666. 10.1016/j.pharep.2017.02.005 28527877

[41] T. Waseem , M. Duxbury , H. Ito , S. W. Ashley , M. K. Robinson , Surgery 2008, 143, 334. 18291254 10.1016/j.surg.2007.09.039 PMC2278045

[42] X. Ye , Z. Liu , H. W. Han , J. Y. Noh , Z. Shen , D. M. Kim , H. Wang , H. Guo , J. Ballard , A. Golovko , B. Morpurgo , Y. Sun , Genes 2023, 14, 1455. 37510359 10.3390/genes14071455 PMC10378756

[43] S. Ezquerro , F. Mocha , G. Frühbeck , R. Guzmán‐Ruiz , V. Valentí , C. Mugueta , S. Becerril , V. Catalán , J. Gómez‐Ambrosi , C. Silva , J. Salvador , I. Colina , M. M. Malagón , A. Rodríguez , The J. Clin. Endocrinol. Metabol. 2019, 104, 21. 10.1210/jc.2018-01171 30137403

[44] C. Gauna , P. J. D. Delhanty , M. O. van Aken , J. A. M. J. L. Janssen , A. P. N. Themmen , L. J. Hofland , M. Culler , F. Broglio , E. Ghigo , A. J. van der Lely , Mol. Cell. Endocrinol. 2006, 251, 103. 16647196 10.1016/j.mce.2006.03.040

[45] H. Yan , W. Yang , F. Zhou , X. Li , Q. Pan , Z. Shen , G. Han , A. Newell‐Fugate , Y. Tian , R. Majeti , W. Liu , Y. Xu , C. Wu , K. Allred , C. Allred , Y. Sun , S. Guo , Diabetes 2019, 68, 291. 30487265 10.2337/db18-0638 PMC6341301

[46] X. Zhang , R. Goncalves , D. M. Mosser , Curr. Protocol. Immunol. 2008, 83, 1. 10.1002/0471142735.im1401s83 PMC2834554 19016445

[47] J. Weischenfeldt , B. Porse , Cold Spring Harbor Protocols 2008, 12, prot5080.

[48] H. Jiang , L. Betancourt , R. G. Smith , Mol. Endocrinol. 2006, 20, 1772. 16601073 10.1210/me.2005-0084

[49] I. Mederacke , D. H. Dapito , S. Affò , H. Uchinami , R. F. Schwabe , Nat. Protoc. 2015, 10, 305. 25612230 10.1038/nprot.2015.017 PMC4681437

[50] J. J. Cai , scGEAToolbox: a Matlab Toolbox for Single‐Cell RNA Sequencing Data Analysis, Oxford University Press, Oxford 2020. 10.1093/bioinformatics/btz830 31697351

