# Supplementary material for: GHSR‐Foxo1 Signaling in Macrophages Promotes Liver Fibrosis via Inflammatory Response and Hepatic Stellate Cell Activation
Source: Adv Sci (Weinh). 2025 Jun 6;12(33):e04223. doi: 10.1002/advs.202504223 (PMC12412619; doi:10.1002/advs.202504223)
Supplement: Supplementary file 1 — Supporting Information [file ADVS-12-e04223-s001.docx]

Supporting Information

**GHSR-Foxo1 Signaling in Macrophages Promotes Liver Fibrosis via Inflammatory Response and Hepatic Stellate Cell Activation**

*Da Mi Kim, Quan Pan, Zeyu Liu, Weiqi Ai, Hye Won Han, Sakhila K. Banu, Robert Y.L. Tsai, Gus A Wright, Shaodong Guo****^*^****, Yuxiang Sun****^*^***


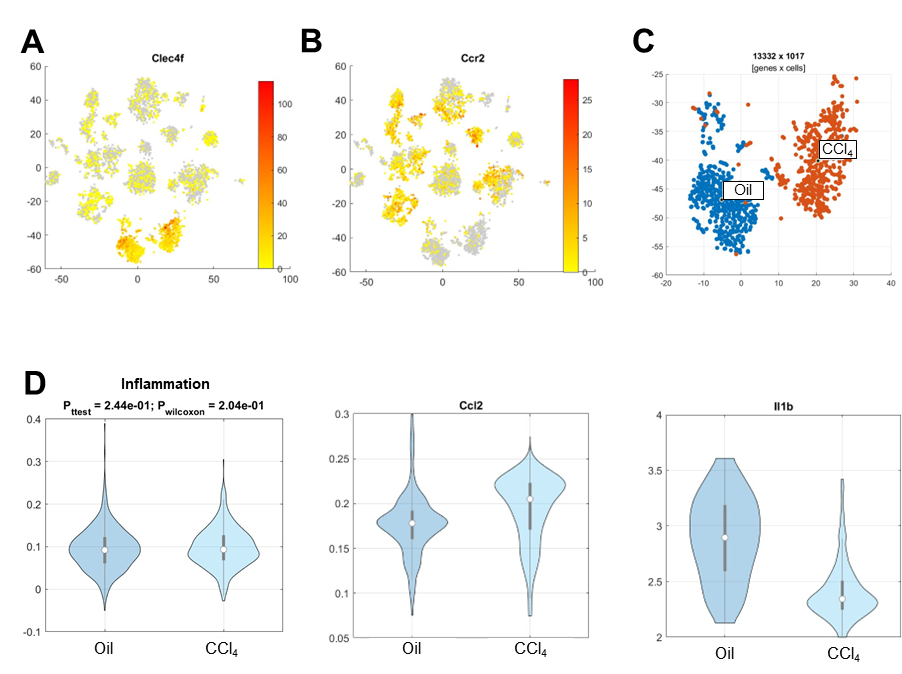


**Figure S1. Macrophage population distribution in fibrotic livers.** scRNAseq analysis of macrophages isolated from control (oil) and fibrotic (CCl_4_) livers. (A and B) t-SNE visualization of the expression of MDM marker *Ccr2* and KC marker *Clec4f*. (C) t-SNE visualization of KCs. (D) Violin plot of inflammation score, *Ccl2* and *Il1b* expression in KC from control or fibrotic livers.

**Table S1. Predicted ligand-receptor interactions from macrophage to HSC.**

| **direction** | **ligand** | **receptor** | **dist** | **correspondence** | **p_val** | **enriched_rank** |
| --- | --- | --- | --- | --- | --- | --- |
| KC/MDMs -> HSCs | CXCL12 | GNAI2 | 6.35E-05 | 19.056864 | 0.000387053 | 1 |
| KC/MDMs -> HSCs | CXCL12 | ITGB1 | 6.35E-05 | 15.097024 | 0.00038754 | 2 |
| KC/MDMs -> HSCs | TGFB1 | CSF1 | 7.92E-05 | 10.723794 | 0.000536973 | 3 |
| KC/MDMs -> HSCs | IL1B | CSF1 | 0.00013 | 17.655235 | 0.000951029 | 4 |
| KC/MDMs -> HSCs | PTPN6 | IFNGR1 | 0.000163 | 11.241235 | 0.001191729 | 5 |
| KC/MDMs -> HSCs | APOA2 | LRP1 | 0.000217 | 26.294172 | 0.001581475 | 6 |
| KC/MDMs -> HSCs | TGFB1 | P4HB | 0.000268 | 13.081883 | 0.001983763 | 7 |
| KC/MDMs -> HSCs | TGFB1 | SDC2 | 0.000287 | 9.859812 | 0.002137899 | 8 |
| KC/MDMs -> HSCs | PTPN6 | IFNGR2 | 0.0003 | 7.937007 | 0.002233028 | 9 |
| KC/MDMs -> HSCs | CALR | P4HB | 0.000305 | 13.461802 | 0.002261709 | 10 |
| KC/MDMs -> HSCs | ICAM1 | VCAM1 | 0.000309 | 8.508833 | 0.002287333 | 11 |
| KC/MDMs -> HSCs | GRN | TNFRSF1B | 0.000361 | 37.184135 | 0.00269917 | 12 |
| KC/MDMs -> HSCs | TGFB1 | TNFRSF11B | 0.000368 | 11.920254 | 0.002748472 | 13 |
| KC/MDMs -> HSCs | PF4 | LRP1 | 0.000426 | 23.205011 | 0.003184821 | 14 |
| KC/MDMs -> HSCs | PF4 | FGFR2 | 0.000426 | 18.457735 | 0.003185704 | 15 |
| KC/MDMs -> HSCs | PF4 | GNAI2 | 0.000426 | 16.850563 | 0.003186101 | 16 |
| KC/MDMs -> HSCs | APOE | SDC2 | 0.0005 | 159.81003 | 0.003782037 | 17 |
| KC/MDMs -> HSCs | IGF1 | EPHA7 | 0.000529 | 9.865246 | 0.004007882 | 18 |
| KC/MDMs -> HSCs | DCN | TGFB1 | 0.000536 | 16.581266 | 0.00404123 | 19 |
| KC/MDMs -> HSCs | YBX1 | TGFB1 | 0.000541 | 14.485394 | 0.004078128 | 20 |
| KC/MDMs -> HSCs | LUM | ITGB1 | 0.000554 | 12.391091 | 0.004192088 | 21 |
| KC/MDMs -> HSCs | PTPN6 | GHR | 0.000571 | 8.8341675 | 0.004314427 | 22 |
| KC/MDMs -> HSCs | LGALS1 | ITGB1 | 0.000573 | 13.086129 | 0.004333913 | 23 |
| KC/MDMs -> HSCs | IGF1 | AXL | 0.000596 | 16.80779 | 0.004543546 | 24 |
| KC/MDMs -> HSCs | C4B | C3 | 0.000712 | 17.209646 | 0.005531571 | 25 |
| KC/MDMs -> HSCs | IGF1 | DDR2 | 0.000737 | 10.482964 | 0.005765126 | 26 |
| KC/MDMs -> HSCs | TNFRSF1B | TNFRSF1A | 0.000751 | 12.3882 | 0.005896939 | 27 |
| KC/MDMs -> HSCs | VCAM1 | ITGA9 | 0.000788 | 11.09686 | 0.00626054 | 28 |
| KC/MDMs -> HSCs | TGFB1 | TGFBR3 | 0.000811 | 12.694817 | 0.006482038 | 29 |
| KC/MDMs -> HSCs | ARF6 | SMAP1 | 0.0009 | 5.406055 | 0.00728222 | 30 |
| KC/MDMs -> HSCs | COPA | CD74 | 0.000941 | 9.907875 | 0.007602467 | 31 |
| KC/MDMs -> HSCs | LGALS9 | LRP1 | 0.000979 | 17.349361 | 0.00796777 | 32 |
| KC/MDMs -> HSCs | LGALS9 | CD47 | 0.000979 | 10.766529 | 0.007969235 | 33 |
| KC/MDMs -> HSCs | IGF1 | CSF1R | 0.000993 | 11.900085 | 0.008103312 | 34 |
| KC/MDMs -> HSCs | YBX1 | NOTCH1 | 0.001006 | 10.891621 | 0.00823443 | 35 |
| KC/MDMs -> HSCs | TGFB1 | THBS1 | 0.001059 | 13.237341 | 0.008777669 | 36 |
| KC/MDMs -> HSCs | CALR | PDIA3 | 0.00108 | 21.352583 | 0.00901076 | 37 |
| KC/MDMs -> HSCs | IGF1 | IGF1R | 0.001108 | 9.380404 | 0.009289187 | 38 |
| KC/MDMs -> HSCs | TLN1 | ITGB5 | 0.001126 | 13.213245 | 0.009443873 | 39 |
| KC/MDMs -> HSCs | TLN1 | ITGB1 | 0.001154 | 26.06065 | 0.009754993 | 40 |
| KC/MDMs -> HSCs | MIF | CD74 | 0.001184 | 28.11434 | 0.010109989 | 41 |
| KC/MDMs -> HSCs | PTPN6 | LIFR | 0.001198 | 17.257425 | 0.010288098 | 42 |
| KC/MDMs -> HSCs | TGFB1 | ITGB5 | 0.001208 | 14.609583 | 0.010412736 | 43 |
| KC/MDMs -> HSCs | TGFB1 | ITGB3 | 0.001231 | 7.1380515 | 0.010690711 | 44 |
| KC/MDMs -> HSCs | TGFB1 | ITGB1 | 0.001235 | 28.814663 | 0.010742312 | 45 |
| KC/MDMs -> HSCs | TGFB1 | PDGFRA | 0.001235 | 30.292356 | 0.010743111 | 46 |
| KC/MDMs -> HSCs | TGFB1 | APP | 0.001235 | 49.287704 | 0.010743302 | 47 |
| KC/MDMs -> HSCs | TGFB1 | CXCL12 | 0.001235 | 162.12672 | 0.010744431 | 48 |
| KC/MDMs -> HSCs | APOE | LRP4 | 0.001254 | 112.94246 | 0.011039202 | 49 |
| KC/MDMs -> HSCs | PTPN6 | IL10RB | 0.001261 | 12.937875 | 0.011125167 | 50 |
| KC/MDMs -> HSCs | CALR | LRP1 | 0.001272 | 51.543503 | 0.01127031 | 51 |
| KC/MDMs -> HSCs | TLN1 | ITGB3 | 0.001312 | 6.455819 | 0.011775151 | 52 |
| KC/MDMs -> HSCs | PTPN6 | IL6ST | 0.001333 | 34.115093 | 0.012033333 | 53 |
| KC/MDMs -> HSCs | PTPN6 | IFNAR2 | 0.001334 | 28.023579 | 0.012046426 | 54 |
| KC/MDMs -> HSCs | IL1B | IL1R1 | 0.001336 | 9.501981 | 0.012074087 | 55 |
| KC/MDMs -> HSCs | VCAM1 | ITGB1 | 0.001351 | 29.001541 | 0.012314058 | 56 |
| KC/MDMs -> HSCs | RAC1 | TLN1 | 0.001351 | 16.364902 | 0.012320908 | 57 |
| KC/MDMs -> HSCs | PTPN6 | IL13RA1 | 0.001366 | 6.4180255 | 0.012549014 | 58 |
| KC/MDMs -> HSCs | IGF1 | PDGFRB | 0.001367 | 34.383144 | 0.012575975 | 59 |
| KC/MDMs -> HSCs | IGF1 | RYK | 0.001368 | 27.11044 | 0.012596341 | 60 |
| KC/MDMs -> HSCs | IGF1 | PDGFRA | 0.001368 | 40.06036 | 0.012599485 | 61 |
| KC/MDMs -> HSCs | IGF1 | APP | 0.001368 | 65.18091 | 0.012599758 | 62 |
| KC/MDMs -> HSCs | IGF1 | FGFR2 | 0.001368 | 52.688934 | 0.012600038 | 63 |
| KC/MDMs -> HSCs | PF4 | SDC2 | 0.001373 | 4.567825 | 0.012686264 | 64 |
| KC/MDMs -> HSCs | RAC1 | PDGFRB | 0.001412 | 39.713425 | 0.013393796 | 65 |
| KC/MDMs -> HSCs | RAC1 | ITGB1 | 0.001413 | 44.013622 | 0.013410021 | 66 |
| KC/MDMs -> HSCs | FAM3C | LAMP1 | 0.001437 | 11.093587 | 0.013969671 | 67 |
| KC/MDMs -> HSCs | LGALS3BP | ITGB1 | 0.00144 | 50.924686 | 0.014078222 | 68 |
| KC/MDMs -> HSCs | GRN | TNFRSF1A | 0.001444 | 76.20326 | 0.014267901 | 69 |
| KC/MDMs -> HSCs | APOE | APP | 0.001448 | 798.8661 | 0.01484926 | 70 |
| KC/MDMs -> HSCs | APOE | LRP1 | 0.001448 | 811.8511 | 0.01496161 | 71 |
| KC/MDMs -> HSCs | TGFB1 | TGFBR2 | 0.00145 | 6.7564163 | 0.015003453 | 72 |
| KC/MDMs -> HSCs | TGFB1 | GLG1 | 0.001452 | 5.9936323 | 0.01502624 | 73 |
| KC/MDMs -> HSCs | RELN | ITGB1 | 0.001458 | 8.219758 | 0.015083388 | 74 |
| KC/MDMs -> HSCs | APOE | LRP5 | 0.001481 | 63.042156 | 0.015333489 | 75 |
| KC/MDMs -> HSCs | RELN | ITGB5 | 0.001485 | 4.167574 | 0.015378468 | 76 |
| KC/MDMs -> HSCs | FAM3C | LIFR | 0.001573 | 3.8995109 | 0.016204494 | 77 |
| KC/MDMs -> HSCs | PTPN6 | SIRPA | 0.001611 | 6.9004455 | 0.016543942 | 78 |
| KC/MDMs -> HSCs | RELN | ITGA8 | 0.001621 | 3.6613972 | 0.016625 | 79 |
| KC/MDMs -> HSCs | RAC1 | ITGA1 | 0.001788 | 8.03229 | 0.018272604 | 80 |
| KC/MDMs -> HSCs | TGFB1 | VASN | 0.001824 | 5.6730046 | 0.018639755 | 81 |
| KC/MDMs -> HSCs | PTPN6 | IL3RA | 0.001838 | 5.2801642 | 0.018815269 | 82 |
| KC/MDMs -> HSCs | IGF1 | PTK7 | 0.001941 | 5.361177 | 0.019960545 | 83 |
| KC/MDMs -> HSCs | IL1A | CXCL12 | 0.00198 | 33.395008 | 0.020364189 | 84 |
| KC/MDMs -> HSCs | RELN | ITGA9 | 0.002021 | 3.145126 | 0.020817864 | 85 |
| KC/MDMs -> HSCs | TGFB1 | TGFBR1 | 0.002038 | 5.5420866 | 0.021007543 | 86 |
| KC/MDMs -> HSCs | APOA1 | LRP1 | 0.002045 | 11.236094 | 0.021089075 | 87 |
| KC/MDMs -> HSCs | CALR | ITGAV | 0.002074 | 5.510633 | 0.021416948 | 88 |
| KC/MDMs -> HSCs | TGFB1 | ITGAV | 0.002111 | 5.355112 | 0.021841924 | 89 |
| KC/MDMs -> HSCs | MIF | CD44 | 0.002148 | 4.5037665 | 0.022303526 | 90 |
| KC/MDMs -> HSCs | PTPN11 | PDGFRB | 0.002216 | 4.706703 | 0.023133872 | 91 |
| KC/MDMs -> HSCs | IGF1 | EPHA3 | 0.002243 | 5.2875185 | 0.023444998 | 92 |
| KC/MDMs -> HSCs | APOE | LRP6 | 0.002315 | 64.51553 | 0.024389289 | 93 |
| KC/MDMs -> HSCs | ANXA2 | PLAT | 0.002338 | 4.102949 | 0.024720946 | 94 |
| KC/MDMs -> HSCs | EBI3 | IL6ST | 0.002342 | 5.362282 | 0.024788124 | 95 |
| KC/MDMs -> HSCs | IGF1 | EPHB4 | 0.002396 | 5.7174573 | 0.025550388 | 96 |
| KC/MDMs -> HSCs | TNFRSF1A | TNFRSF1B | 0.002399 | 3.8181608 | 0.025587234 | 97 |
| KC/MDMs -> HSCs | IGF1 | MERTK | 0.002449 | 5.3518815 | 0.026243675 | 98 |
| KC/MDMs -> HSCs | CXCL12 | ITGB3 | 0.002519 | 3.7398784 | 0.02726792 | 99 |
| KC/MDMs -> HSCs | LGALS9 | PTPRK | 0.002578 | 3.1556952 | 0.028128829 | 100 |

**Table S2. Key Resources Table.**

| **Antibodies** | **Source** | **Catalog number** |
| --- | --- | --- |
| Mac-2 | Abcam | ab76245 |
| anti-FcγR II/III antibody | BD Bioscience | 553141 |
| eFluor 450 anti-mouse CD45 | eBioscience | 48-0451-82 |
| PerCP anti-mouse Ly6G | BioLegend | 127653 |
| APC-Cy7 anti-mouse CD11b | BioLegend | 101225 |
| Alexa Fluor 488 anti-mouse F4/80 | eBioscience | 53-4801-82 |
| BV750 anti-mouse CD38 | BD bioscience | 747103 |
| BV650 anti-mouse CD206 | BioLegend | 141723 |
| PE-Cy7 anti-mouse TNFα | BioLegend | 506324 |
| PE anti-mouse IL1β | eBioscience | 12-7114-80 |
| PE-Cy5.5 anti-mouse iNOS | eBioscience | 35-5920-80 |
| Alexas Fluor 700 anti-mouse ARG1 | eBioscience | 56-3697-82 |
| p-Foxo1-S273 | Covance | N/A |
| αSMA | Abcam | ab7817 |
| Foxo1 | Cell Signaling Technology | 2880S |
| TGF-β1 | Cell Signaling Technology | 3711S |
| p-CREB | Cell Signaling Technology | 9198S |
| CREB | Cell Signaling Technology | 4820S |
| GAPDH | Cell Signaling Technology | 2118S |
| Β-actin | Cell Signaling Technology | 4970L |
| GHSR | Invitrogen | 720278 |
|  | | |
| **Chemicals, assay kits** | **Source** | **Catalog number** |
| CCl**_4_** | Millipore-Sigma | 319961 |
| live/dead aqua | Thermo Fisher Scientific | L34966 |
| Lipofectamine 3000 | Invitrogen | L3000015 |
| H89 | Millipore-Sigma | 371962 |
| TRIzol reagent | Invitrogen | 15596026 |
| Immunology Multiplex Assay | Millipore-Sigma | MHSTCMAG-70KPMX |
| iScript cDNA synthesis system | Bio-Rad | 1708841 |
| SYBR Green Supermix system | Bio-Rad | 1725274 |
| Free Active TGF-β1 ELISA kit | BioLegend | 437707 |
|  | | |
| **qPCR primer** | **Sequence (Forward)** | **Sequence (Reverse)** |
| Col1a1 | GCGAGTGCTGTGCTTTCTG | GGTCCCTCGACTCCTACATCT |
| Acta2 | ATGAAGCCCAGAGCAAGAGA | ATGTCGTCCAGTTGGTGAT |
| Tnfa | CCACCACGCTCTTCTGTCTA | GGTTTGCTACGACGTGGGC |
| Il1b | TGTTCTTTGAAGTTGACGGACCC | TCATCTCGGAGCCTGTAGTGC |
| Il6 | ACAAGTCCGGAGAGGAGACT | GAATTGCCATTGCACAACTCT |
| Ccl2 | CACTCACCTGCTGCTACTCA | GCTTGGTGACAAAAACTACAGC |
| Tgf1 | ATCCTGTCCAAACTAAGGCTCG | ACCTCTTTAGCATAGTAGTCCGC |
| Foxo1 | AGATGAGTGCCCTGGGCAGC | GATGGACTCCATGTCACAGT |
| Ghsr | AAGATGCTTGCTGTGGTGGT | AGCGCTGAGGTAGAAGAGGA |
|  | | |
| **Recombinant DNA** | **Source** | **Catalog number** |
| pcDNA3.1 | Invitrogen | V79020 |
